# Identification of clustered microRNAs using an *ab initio *prediction method

**DOI:** 10.1186/1471-2105-6-267

**Published:** 2005-11-07

**Authors:** Alain Sewer, Nicodème Paul, Pablo Landgraf, Alexei Aravin, Sébastien Pfeffer, Michael J Brownstein, Thomas Tuschl, Erik van Nimwegen, Mihaela Zavolan

**Affiliations:** 1 Biozentrum, Universität Basel, Basel, Switzerland; 2 Laboratory of RNA Molecular Biology, Rockefeller University, New York, USA; 3 J. Craig Venter Institute, Functional Genomics, Rockville, USA; 4 IBMP-CNRS, Strasbourg, France

## Abstract

**Background:**

MicroRNAs (miRNAs) are endogenous 21 to 23-nucleotide RNA molecules that regulate protein-coding gene expression in plants and animals via the RNA interference pathway. Hundreds of them have been identified in the last five years and very recent works indicate that their total number is still larger. Therefore miRNAs gene discovery remains an important aspect of understanding this new and still widely unknown regulation mechanism. Bioinformatics approaches have proved to be very useful toward this goal by guiding the experimental investigations.

**Results:**

In this work we describe our computational method for miRNA prediction and the results of its application to the discovery of novel mammalian miRNAs. We focus on genomic regions around already known miRNAs, in order to exploit the property that miRNAs are occasionally found in clusters. Starting with the known human, mouse and rat miRNAs we analyze 20 kb of flanking genomic regions for the presence of putative precursor miRNAs (pre-miRNAs). Each genome is analyzed separately, allowing us to study the species-specific identity and genome organization of miRNA loci. We only use cross-species comparisons to make conservative estimates of the number of novel miRNAs. Our *ab initio *method predicts between fifty and hundred novel pre-miRNAs for each of the considered species. Around 30% of these already have experimental support in a large set of cloned mammalian small RNAs. The validation rate among predicted cases that are conserved in at least one other species is higher, about 60%, and many of them have not been detected by prediction methods that used cross-species comparisons. A large fraction of the experimentally confirmed predictions correspond to an imprinted locus residing on chromosome 14 in human, 12 in mouse and 6 in rat. Our computational tool can be accessed on the world-wide-web.

**Conclusion:**

Our results show that the assumption that many miRNAs occur in clusters is fruitful for the discovery of novel miRNAs. Additionally we show that although the overall miRNA content in the observed clusters is very similar across the three considered species, the internal organization of the clusters changes in evolution.

## Background

MicroRNAs (miRNAs) form a recently-discovered family of single-stranded RNA molecules of length approximatively 22 nucleotides that are present in all higher eukaryotes [1,2]. As shown by the growing number of specific examples, they regulate gene expression at a post-transcriptional level by binding to specific mRNA targets whose translation is thereby inhibited [3]. Although some details of miRNA biogenesis are still missing, a consensus scenario has now emerged: primary miRNA (pri-miRNAs) are transcribed generally by polymerase II [4], these transcripts are processed in the nucleus by the Drosha endonuclease [5] and exported as individual pre-miRNA stem loops to the cytoplasm by Exportin 5 [6]. In the cytoplasm, the mature forms are produced through the action of the Dicer endonuclease [7]. It appears that a crucial feature throughout these processing steps is a stem loop secondary structure [8].

An upper bound on the number of miRNAs present in the human genome was initially set by Lim *et al. *to a few hundred [9]. Recently however, this number has been re-evaluated by Berezikov *et al. *who argued that mammalian genomes encode close to a thousand miRNAs [10]. Thus, the debate about the number and identity of the miRNAs in mammalian genomes is open, especially considering that these estimates concern only miRNAs that are conserved between relatively distant species such as primates and rodents and not miRNAs that are of a more recent evolutionary origin.

The complete miRNA transcription units (pri-miRNA) remain to be defined, although some studies have already associated miRNAs with cDNAs sequences corresponding presumably to pri-miRNAs that can be found in sequence databases [11]. This and other studies (as well as our own unpublished data) show that some miRNAs are transcribed as polycistronic transcripts which are several kb long. Additional support for this hypothesis comes from a recent study that revealed that miRNAs that are found within 50 kb of each other on the same strand display correlated expression in microarray experiments [12]. Therefore the genomic regions around the loci of known miRNAs appear particularly promising for discovering additional miRNAs.

In the past few years several algorithms have been designed for detecting (pre-)miRNAs, and they proved to be extremely efficient in supporting experimental mature miRNAs discovery [10,13-15]. Very generally, these methods identify specific secondary structures corresponding to miRNA precursors in regions of the genome that are conserved between species. Recent experiments have uncovered, however, a number of miRNAs that do not have close homologs in the sequenced genomes available to date, such as for example the miRNAs encoded by the Epstein-Barr virus (EBV) [16]. This finding emphasized that it would be desirable to have a method able to predict miRNAs in a single genome, without an absolute requirement for cross-species conservation. We developed such a prediction method and we used it to discover miRNAs in a number of members of the herpes virus family [17]. By similarity with protein-coding gene prediction methods that only scan genomic regions looking for signals characteristic to protein-coding genes and do not use external transcripts or other genomes, we called our method *ab initio*.

Here we apply our method to search for novel miRNAs that are in close proximity, and may be co-transcribed, with already known miRNAs. As the set of known miRNAs we take the human, mouse and rat sequences from the April 2005 release of the Rfam miRNA repository [18]. To evaluate the performance of the method, we use a growing set of mammalian sequences that are cloned in the Tuschl laboratory [19]. In the following we first present the general ideas behind our pre-miRNA prediction method, then show the results of two validation tests and finally move on to its application to the discovery of potentially co-transcribed miRNAs in human, mouse and rat.

## Results

### Overview of the pre-miRNA prediction method

The general idea of our approach to pre-miRNA prediction is to design a computational method that can be used to better understand the constraints that define miRNA precursors in relationship to their processing enzymes. We start with the observation that one of the generic features shared by all miRNA genes is the secondary structure assumed by the transcript region surrounding the mature miRNA. Indeed, mature miRNAs appear to reside inside one arm (5' or 3') of a stem loop with good, though not perfect, base pairing during the various steps of the biogenesis [1,2]. This stem loop structure is important for miRNA precursor recognition by RNAase III enzymes Drosha [5] and Dicer [7] as well for the export of the miRNA precursor from the nucleus [6]. The intermediates in this processing pathway differ in the length of the sequence surrounding the mature miRNA, implying that the stem loop structure of the pre-miRNA persists independently of the precise sequence context that varies from several kb for the pri-miRNA transcripts to 50–70 nucleotides for the relatively short pre-miRNA. We thus design our prediction method to identify such "context-robust" (or shortly, "robust") stem loops and then characterize their compositional and secondary structure properties in relationship to those of known pre-miRNA as well as negative examples. Our approach consists in three steps:

1. From the input sequence we first extract a set of genomic regions that are predicted to exhibit the same stem loop secondary structure, irrespectively of the size of the larger transcript in which they are embedded. We call these "robust" stem loops.

2. Each of the stem loops thus detected is assigned a score that reflects its similarity to known cases of human pre-miRNAs. Since the precise structural features that contribute to miRNA precursor recognition by processing enzymes are not known, we use a device from statistical machine learning called "support vector machine" (SVM), as follows. We describe any given stem loop in terms of sequence and structure features. Then, using two training sets consisting of known human miRNA precursor sequences as positive examples and random subsequences from genomic regions, tRNA, rRNA and mRNA genes as negative examples we build a model which describes the relative contribution (weight) of each of the features to the score assigned to any given stem loop. The score measures the distance (in our feature space) from the candidate stem loop to the hypersurface that best separates the positive from the negative examples. The weights associated with the features that we used to describe the stem loops give us insight into the constraints that appear to be most important for the recognition of miRNA precursors by the processing enzymes.

3. In order to guide experimental investigations, we develop a probabilistic mathematical framework that enables us to estimate the pre-miRNA content of the input genomic sequence from the scores assigned to all the stem loops identified in this sequence. This framework that has the advantage of not being dependent of the somehow arbitrary score cut-off used to define the predicted miRNA precursor stem loops.

Further details about each of these components are presented in the "Methods" section.

### Validation of the method

#### Identification of viral miRNAs

**Figure 1:**
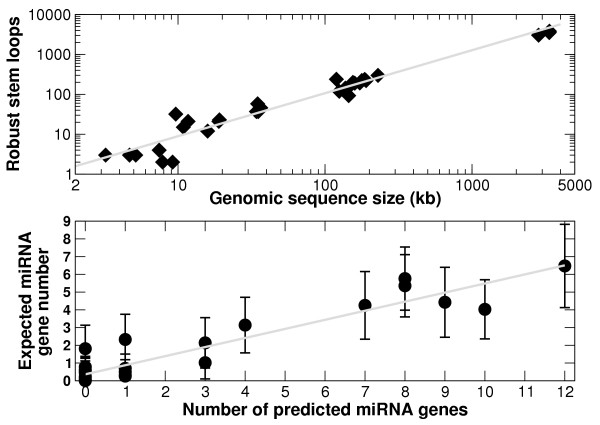
**Robust stem loops and pre-miRNA predictions**. The upper panel shows a plot of the number of robust stem loops versus the size of the genomic sequence they originate from. The data come from the application of our method to viruses [17] and, for the three at the extreme right-hand side, from the present study (see Subsection "Identification of novel clustered miRNAs"). The linear dependence is very clear and corresponds to an average of 1.2 robust stem loop every kb. The lower panel shows the relation between the number of predicted pre-miRNAs (stem loops with positive SVM score) and the expected number of pre-miRNAs, given by Equation 4. The linear dependence is also clear here, but the slope is smaller than 1, which would have corresponded to a strict equality between the two sets.

We initially validated our method on a set of eight human pathogenic viruses for which experimental investigations were simultaneously undertaken [17]. We made 32 pre-miRNA predictions, out of which 13 were confirmed by the cloning study, giving a confirmation rate of 41%. As explained in the "Method" section, the first step of our prediction method consists in extracting genomic regions which are able to form robust stem loop structures. The number of such regions grows linearly with the genome size (Figure 1, upper panel). The number of predicted pre-miRNAs, however, is not merely a linearly function of the genome size [17]. This property shows that the classifier that we have developed captures specific features of pre-miRNAs that are not uniformly distributed across the input genomic sequence. We have also developed a method for estimating the expected number of pre-miRNAs in a given genomic sequence (see Equation 4 from the "Methods" section). As shown in the lower panel of Figure 1, the expected number of pre-miRNAs in a genome is, as expected, strongly correlated with the number of predicted pre-miRNAs (i.e. the number of stem loops with a positive prediction score). At the same time this statistics is more robust against small variations in the value of the score threshold defining the predictions. An additional advantage of using this estimation approach is that it enables us to identify genomic regions that are likely to give rise to miRNAs without having to pinpoint their precise location. This is useful for directing experimental studies to promising genomes or genomic regions.

#### Recovering known miRNA in distantly-related species

It is perhaps not surprising that we can predict viral miRNAs, given that they are processed by the miRNA processing machinery of the human host, as are the miRNAs with which we have trained our model. To test the ability of our method to identify pre-miRNAs in distantly-related species, we applied it to the regions known to encode miRNA loci in the invertebrate *Caernorhabditis elegans*. Of the 116 known miRNA precursors, we recover 50 (43%) as predictions. This results indicates that our prediction method has a reasonable sensitivity for a wide range of genomes from worms, to vertebrates to animal viruses.

### Predictions: clustered miRNAs in human, mouse and rat

#### Identification of novel clustered miRNAs

miRNAs are often found in genomic clusters, some of which are believed to be transcribed as a single transcript (polycistronic pri-miRNA). A nice example is the cluster of hsa-mir-17, whose elements reside within a 1 kb interval on human chromosome 13 and are indeed co-transcribed (cDNA Genbank accession number BC040320). Motivated by the recent microarray study by Baskerville and Bartel showing that correlated expression of miRNAs can be shown up to the order of 50 kb of relative genomic distance [12], we set to discover novel miRNAs in the neighborhood of confirmed miRNAs from the Rfam database [18]. We adopt the following strategy. We first group into clusters the known miRNAs from human/mouse/rat Rfam6.0 that are closer than 10 kb from each other and have the same transcription sense. We find that 105/111/82 of the total 227/232/186 pre-miRNAs (i.e. 46%/48%/44%) belong to a cluster of at least two elements. By adding these to the miRNAs that so far appear to be isolated, we obtain a total of 162/161/138 clusters. We then extend the genomic regions of all of these clusters by 10 kb on each side and submit the resulting sequences to the prediction method. In total, we analyze 3.36/3.35/2.84 million nucleotides and we find that 224/192/208 of the 3829/3537/3034 candidate stems are classified as predicted pre-miRNA by our prediction method. After filtering out the known pre-miRNAs in these clusters, we obtain a total of 89/66/105 predictions, given in the Additional files 1, 2, and 3.

In order to validate these predictions, we have searched a large database of small RNAs from human, mouse and rat that have been cloned in the Tuschl lab [19]. We consider a prediction to be validated if one of the arms of the stem loop matches *perfectly *a cloned small RNA that is not known to be derived from a rRNA, tRNA, snRNA or snoRNA. Additionally, although our predictions include sequences coming from repeated regions, we discard cases where the cloned small RNA has more than two perfect mappings to the genome of the species it originates from. We then find that 20/17/6 of the predictions have a match from the same species, and these numbers raise to 22/20/26 if matches from small RNAs from all three species are allowed. This corresponds to confirmation rates between 25% and 30%, which are somewhat lower than the one obtained with the earlier application of our method to viruses (40%). The pre-miRNAs predicted in repetitive elements are partially responsible for these lower confirmation rates. The complete list of the confirmed predictions together with the sequences of the cloned miRNAs are given in the Additional files 4, 5, and 6.

The false negative rate of our prediction method, as determined from the cloning data, is 34%, a value which is close to the false negative rate of 29% that we obtained for our SVM training set, using a threshold score of 0. This indicates that the prediction method behaves as expected. For completeness, the false negative predictions are shown in the Additional files 7, 8, and 9.

If we consider our results at the level of genomic clusters, we find 5 novel clusters in human, 5 in mouse and 6 in rat. By "novel clusters" we mean a set of precursor miRNAs that contains, beside the confirmed predictions, known cases that were not considered to be in clusters, i.e. which did not have another known pre-miRNA with same transcription sense at distance smaller than 10 kb from its genomic location. This corresponds to an increase of the total number of clusters from 40/40/34 to 44/45/38 in human/mouse/rat.

#### Phylogenetic conservation of the clustered miRNAs

Since cross-species conservation was not used in the process of generating our predictions, we can now go back and ask the question of whether the predicted pre-miRNAs are indeed conserved between human, mouse and rat. We define the "conservation" relation between two given species at three progressive levels, corresponding to the three columns labeled "Conservation" in the Additional files 1, 2, and 3. The first level requires that a homologous sequence is found for a predicted miRNA precursor in another genome (sequences alignment *E*-value given by the WU-Blast program ≤ 10^-5^). The second level requires that both the predicted precursor as well as the homologous sequence fold into simple (not branched) stem loops. The third level requires that the two homologous sequences are predicted to be miRNA precursors by a method that uses cross-species conservation. For this purpose, we use the web interface to the MiRscan method with the default parameters (threshold at score value 14) [13].

As shown in the Additional files 1, 2, and 3, the predictions that are conserved across species are more likely to be experimentally confirmed than the one that are not. Indeed the confirmation rates are 1%, 22% and 49% for predicted precursors with homologs in none, precisely one and both species, respectively. We thus conclude that the cross-species information strongly improves the specificity of the prediction method. If we now restrict ourselves to predictions that have experimental confirmation, we find that for almost all of them (95%) a strong sequence homology is equivalent to a conserved stem loop structure. Additionally, we learn from the third conservation column that only 68% of the conserved and confirmed miRNAs that we predict are also classified as miRNA precursors by MiRscan.

In some cases we discovered miRNAs that are known in some species, but were not reported in others. This is the case of the rat homolog of mmu-mir-1, which corresponds to our predicted RP-79. The predicted precursor has over 97% identity relative to the mouse precursor, and the region corresponding to the mature miRNA is perfectly conserved. This miRNA has not been cloned in rat. In other cases we discovered miRNAs that are conserved across all three species but that are found in the neighborhood of a miRNA only known to exist in one of the species. This is the case of RP-97, which is close to rno-mir-421. For this miRNA we found cloning and conservation evidence in mouse and human as well. Note, however, that we do not report here candidate pre-miRNAs that are homologous to some of our predictions but that were not part of our predicted set because in their species of origin they are not found in the neighborhood of a known miRNA.

#### Genomic locations of the miRNA clusters

**Figure 2:**
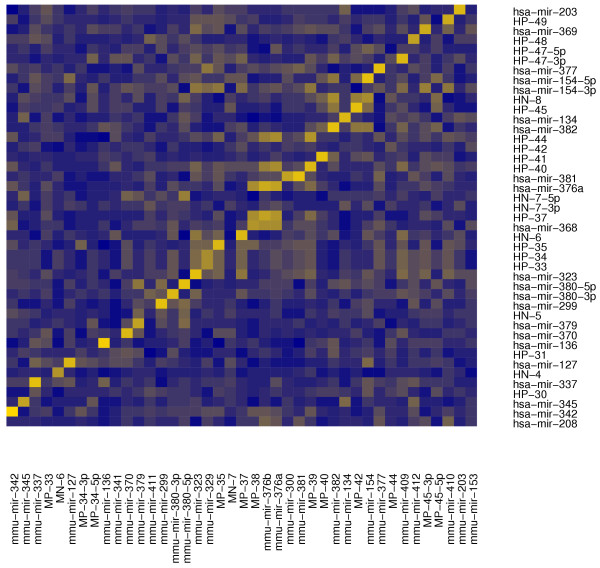
**Mappings between the human and mouse imprinted loci**. For human and mouse we take all the sequences of the mature miRNAs belonging to the imprinted loci on chromosomes 14 and 12 that are present in Rfam6.0, in our set of confirmed predictions (Additional files 4 and 5) and in the false negatives set (Additional files 7 and 8). We sort them according to their genomic coordinates and display on the graph the sequence similarity (i.e. the number of matches in a CLUSTALW alignment) for all possible pairs between the two set. Although a clear diagonal signal indicating cross-species conservation is visible, it is also very clear that species-specific features altering it are numerous, as described in the text.

**Figure 3:**
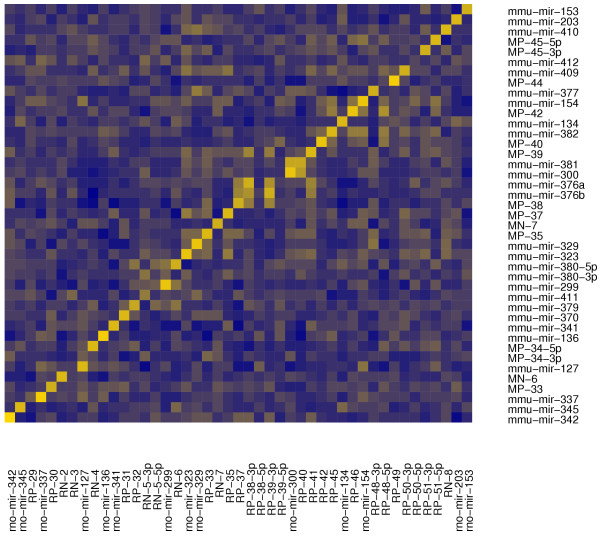
**Mappings between the human and mouse imprinted loci**. The same as Figure 2, but for mouse and rat. We clearly observe a much better diagonal signal, as expected. Off-diagonal bright spots indicate miRNAs that are related in sequence and that have probably arisen by duplication of a common ancestor.

Most of the confirmed predictions come from a relatively small number of clusters. These are the following:

1. The orthologous loci located on chromosome 14 in human, chromosome 12 in mouse and chromosome 6 in rat, each of which is less than 200 kb in length. The human locus has been shown to be imprinted [20]. Only a few miRNAs from these loci have been deposited in Rfam, although other studies have also published computational predictions matching them [10,20,21]. The fine-grained structure of these loci has some species-specific aspects, as illustrated by Figures 2 and 3. The figures show all the validated miRNAs in these regions, including those with suboptimal prediction scores from the Additional files 7, 8, and 9. We find that some miRNAs that are related in sequence, and have presumably arisen by duplication (such as the mir-368/mir-376-related sequences) have different numbers of copies in rodents and human. We also find rodent- (MP-33/RP-30, MP-34/RN-4, MP-44/RP-49) and human-specific (HP-31) miRNAs, meaning that they do not have very close mature form homologs in the other species. Yet in other cases the human and mouse sequence have diverged slightly, as the predicted miRNAs HP-30/MP-32 and HP-41/MP-41. Because the mature forms of these miRNAs differ by a deletion or a substitution, and because we only considered perfectly matching small RNAs from human as validation, only the human miRNA genes end up being validated (see the Additional files 1 and 2). Finally, there are cases of more complicated species-specific evolutionary pattern. For example, mir-329 appears to have diverged between human and mouse: at the syntenic location in human we find two identical copies of a miRNA distantly-related to mmu-mir-329 (HP-33 and HP-34). Furthermore, this cluster has a complex composition, containing other related sequences {hsa-mir-323, HP-33, HP-34, HP-35, HN-6} whereas the corresponding mouse cluster {mmu-mir-323, mmu-mir-329, MP-35, MN-7, MP-37} additionally contains a rodent-specific sequence, MN-7, which is not related to the other sequences in the cluster. A similar situation can be found in the human cluster {hsa-mir-368, HP-37, HN-7, hsa-mir-376a} which corresponds to the mouse {mmu-mir-376a, mmu-mir-376b, MP-38}.

2. Chromosome X also contains a substantial number of novel miRNAs in all three species. They are spread over the full chromosome and eventually form small clusters with only a few elements. In the mir-17 cluster paralog on chromosome X, whose evolution has been analyzed in detail by Tanzer and Stadler [22], we found two additional miRNAs that are conserved in all three species. In the order of genome location, the cluster then reads: mir-106a, HN-14/MP-56/RP-100, mir-19b-2, mir-92-2, and HP-85/MN-8/RP-99. Consistent with the evolutionary scenario proposed by these authors we find that these novel miRNAs are relatively close in sequence to other miRNAs in the cluster. For instance the mature miRNA sequence of MP-56 has only two mismatches with mmu-mir-17, mmu-mir-20 and mmu-mir-106a. We observe a similar situation for another cluster on human chromosome X: hsa-mir-188, HN-11, HP-77, HN-12, and HN-13 (in transcription sense order). Whereas only HP-77 is a confirmed prediction and possesses a close homolog in mouse (MN-9), this cluster contains further three related miRNAs that have negative scores but that have been confirmed experimentally.

3. Apart from the above mentioned miRNAs, we found a few other cases of clustered, and potentially co-transcribed miRNAs: two in human (chromosomes 16 and 17), four in mouse (chromosomes 3, 10, 11 and Un-random) and three in rat (chromosomes 9, 10 and 18).

### Comparison with other prediction methods

To evaluate the performance of our approach relatively to other large-scale miRNA prediction methods, we perform the following test. We take the (pre-)miRNA predictions sets provided by the most extensive predictions studies in the recent past [10,21,23]. Since these results have been published at different times and some of the predictions sets contain also known miRNAs, we set the Rfam6.0 release as the reference set of known miRNAs. The set of miRNAs used to perform sensitivity/specificity tests contains those that have been introduced in the most recent version of the miRNA repository, Rfam7.0 and that only became available while our manuscript was under revision, as well as those (pre-)miRNAs that are predicted by any of the four methods (including ours) and that are confirmed by the cloning data [19]. This set comprises 38 miRNAs. Table 1 shows that, when tested on miRNAs that are conserved between human and mouse or human and rat, our method has comparable performance to methods that make predictions from conserved genomic regions. Overall, we have somewhat higher sensitivity, at the expense of somewhat lower specificity compared to the methods of Berezikov *et al. *and Xie *et al.*. Interestingly, each of four methods is able to predict some (pre-)miRNAs that are not predicted by any of the other ones.

**Table 1:** Sensitivity and specificity. This table shows a comparison of the performances of various methods when applied to the genomic loci considered in this work. The sensitivity was calculated by taking the union of the miRNAs predicted by any method but confirmed experimentally. This set contains 38 elements.

	Altuvia *et al. *[23]	Berezikov *et al. *[10]	Xie *et al. *[21]	Our method
Conserved predictions in analyzed loci	87	179	29	36
Predictions not in Rfam 6.0	78	27	23	36
Predictions in Rfam 7.0 or in cloning set [19]	18	21	20	23
Sensitivity	47%	55%	53%	64%
Specificity	23%	77%	87%	64%
Uniquely predicted pre-miRNAs	4	4	1	4
Overlap of our predictions with others	10	14	14	-

## Discussion

We have developed a computational method for *ab initio *prediction of precursor miRNAs that we applied here to the problem of identifying clustered, probably co-transcribed miRNAs. As explained in details in the "pre-miRNA prediction method" subsection and in the "Methods" section, our approach is based on a mechanistic model for the action of enzymes like Drosha or Dicer, and uses only the information contained in the input sequence and secondary structure. In doing so we neglect important aspects such as the fact that any stem loop that we consider a candidate pre-miRNA has to be be transcribed and accessible to all the processing enzymes responsible for ultimately producing a mature miRNA. This aspect is, in part, responsible for a relatively high number of false positives that we would obtain if we were to run the prediction method on an entire mammalian genome. But by applying the method to regions around loci of already known miRNAs, we believe that we circumvent the issue of whether the genomic regions that we analyze are transcribed and are accessible to all the processing enzymes. Our results (68 novel, experimentally confirmed cases for 260 predictions, or a 26% hit rate) show that this assumption was justified. This is also indicated by another recent study that identified 8 of our 68 predicted and confirmed miRNAs [23]. The given percentage is a lower bound for the performance of our method on these genomic regions, since it cannot be excluded that some of the predicted pre-miRNAs are not detected or are expressed in tissues or developmental stages other than the ones that have been used in the experiments. Furthermore, we did not filter out from our predictions those that fall inside repeat elements and may have higher likelihood of being false positives. Although this inclusion lead to a lower hit rate, we have seen above that this allowed us to discover a rich structure in the imprinted clusters.

Virtually all of precursors of the validated miRNAs have some homolog (albeit somewhat different at the level of the mature miRNA) in at least one other species, and a number of factors may contribute to this effect.

1. We have focused our search on regions that are already known to contain conserved miRNAs and, as can be observed from out comparison of human and mouse loci, miRNAs that are close to each other in the genome are frequently related in sequence.

2. We have used for validation data from all three species, and we have considered a predicted pre-miRNA to be confirmed even in cases in which the supporting small RNA cloning data came from another species.

3. Finally, the mouse and rat genomes are quite close to each other and we therefore expect that almost all of the mouse miRNAs have rat homologs, and vice versa.

Note however that 17 out of the 46 confirmed mouse and rat miRNAs appear to be rodent-specific (i.e. 37%), and one confirmed miRNA appears to be human specific (HP-31). These miRNAs would be difficult to discover using other methods either due to lack of cross-species conservation or because the genomes that are sharing the miRNA are too close.

Although our "ab initio" approach to pre-miRNA discovery was initially designed and successfully applied to detect species-specific miRNAs [17], we find that it retains its value when applied to a situation where cross-species conservation plays a important role. In fact, we were able to discover conserved miRNAs that were missed by three methods that use cross-species comparisons to make their predictions [10,15,21]. Concretely, in the large imprinted clusters from human chromosome 14, mouse chromosome 12 and rat chromosome 6, almost half (48%) of the miRNAs that we predicted and were confirmed experimentally are novel (see the Additional files 1, 2, and 3).

It would be, of course, very instructive to understand what factors contribute to the different results obtained by different prediction methods. Although this is not the topic of our current study, we discuss some of these factors below. Generally, all these approaches, including ours, are based on a two-level strategy: first identify a relatively small set of candidates and then examine these candidates in detail to make predictions.

1. A fundamental difference between the first layer of our method and any cross-species-based analysis is the fact that we have tried to take into account mechanistic considerations rather than evolutionary conservation or statistical properties. That is, by first identifying "robust" stem loops (see "Methods") we not only pick up genomic regions which are likely to form suitable RNA secondary structure (which may be gotten using programs such as *RNALfold *[24]), but we also take into account the fact that the stem loop has to be present in the various stages of the miRNA biogenesis. This implies a dependency of the stem loop secondary structures that we identify on the flanking genomic regions, which should not compete too strongly for pairing with nucleotides within the miRNA precursor and thereby destroying its secondary structure. This mechanism naturally gives a basis to the observation that the functional RNAs, and in particular miRNA precursors, have thermodynamically more stable structures than randomized sequences with the same (di-)nucleotide composition [25] (this property has been used to predict precursor miRNAs [10], see next paragraph). The same property, namely robustness of functional RNA secondary structure elements with respect to varying sequence context, has been described in RNA viruses [26].

2. The second layer of the approach consists in scoring the candidate regions. In our case, these regions are already predicted to form stem loop secondary structures. Our choice of implementing a support vector machine (SVM, see "Methods") to distinguish between "good" and "bad" precursor miRNA candidates has several advantages. First it also includes information about what a miRNA precursor should not be, unlike the MiRscan scoring scheme which is only based on positive examples. Additionally, our procedure enables us to use the positive and negative examples to compute the weights with which the various features in our model should contribute to the score (see Tables 2, 3, 4, and 5). This detailed description of the sequence composition and structural features of the miRNA precursors enables our classifier to perform better than classifiers based simply on the thermodynamic stability of miRNA precursor stem loops [25]: on our training data, the SVM has at least 3-fold lower false positive rate compared to a classifier based on the RANDfold algorithm for any given rate of false negative predictions (data not shown). Additionally, the weights assigned by the SVM for individual features give us a deeper understanding of miRNA processing than procedures that only use statistics of secondary structure stability of pre-miRNAs relative to randomized variants [10].

**Table 2:** SVM features to describe stem loops, part 1. These quantities are calculated over the entire stem loop structure. The weights are normalized with respect to the first feature, which turns out also to be the one with smallest value.

Index	Feature description	SVM weight
1	Free energy of folding	-1
2	Length of the longest simple stem	0.547
3	Length of the hairpin loop	0.193
4	Length of the longest perfect stem	0.030
5	Number of nucleotides in symmetrical loops	-0.006
6	Number of nucleotides in asymmetrical loops	0
7	Average distance between internal loops	-0.029
8	Average size of symmetrical loops	0.207
9	Average size of asymmetrical loops	-0.171
10/11/12/13	Proportion of A/C/G/U nucleotides in the stem	0.005/-0.003/-0.004/0.001
14/15/16	Proportion of A-U/C-G/G-U base pairs in the stem	0.015/-0.002/-0.006

**Table 3:** SVM features to describe stem loops, part 2. These quantities are calculated over the longest symmetrical region of the stem loop, i.e. the longest region without any asymmetrical loop.

Index	Feature description	SVM weight
17	Length	0.353
18	Distance from the hairpin loop	0.126
19	Number of nucleotides involved in internal loops	0.041
20/21/22/23	Proportion of A/C/G/U nucleotides	0.082/0.241/0.078/0.059
24/25/26	Proportion of A-U/C-G/G-U base pairs	0.211/0.254/-0.131

**Table 4:** SVM features to describe stem loops, part 3. These quantities are calculated over the longest region in which the difference between the 5' and 3' components of asymmetrical loops is not larger than Δ*l *(we will call this "relaxed symmetry region").

Index	Feature description	SVM weight
27	Length	0.189
28	Distance from the hairpin loop	0.116
29	Number of nucleotides involved in symmetrical internal loops	-0.220
30	Number of nucleotides involved in asymmetrical internal loops	-0.176
31/32/33/34	Proportion of A/C/G/U nucleotides	0.024/0.077/-0.079/0.149
35/36/37	Proportion of A-U/C-G/G-U base pairs	0.317/0.123/-0.156

**Table 5:** SVM features to describe stem loops, part 4. These quantities are calculated over all windows of length corresponding to miRNA length *l*_*m *_that we could place on the candidate stem loop.

Index	Feature description	SVM weight
38	Maximum number of base pairs	-0.140
39	Minimum number of nucleotides in asymmetrical loops	-0.025
40	Minimum asymmetry over the internal loops in this region	0.026

Adding our confirmed predictions (around 25 per species) to the already known miRNAs from Rfam6.0 (227/232/188 for human/mouse/rat), we have reached the upper bound on the number of miRNAs that was estimated by Lim *et al. *to be around 255 [13]. It is now important to realize that this estimate was based on an assumption of miRNA conservation over an evolutionary distance up to the pufferfish *fugu rubripes*. The more recent estimate of Berezikov *et al.*, with less stringent assumptions about conservation lies in the range of about a thousand miRNAs [10]. From the present work we have learned that these methods have missed some conserved miRNAs. Moreover, some miRNAs are only represented in closely-related species (such as mouse and rat) and there are also families of closely related miRNAs that differ in precise composition across species. All these considerations lead us to conclude that the miRNA discovery is still not completed, and moreover, that hundreds of miRNAs are yet awaiting experimental confirmation.

Although the hypothesis that miRNA loci tend to occur in clusters which are probably co-transcribed has been useful in the discovery of novel miRNAs, there are interesting open questions about the expression of the co-transcribed miRNAs. In particular, not all of the known examples of co-transcribed miRNAs show strongly correlated expression patterns [27]. This indicates that yet unknown processing factors lead to differential expression of the clustered miRNAs by either making the processing enzymes having different efficiencies on different templates or by directing transcription from alternative transcription start sites.

## Conclusion

We have developed a computational method to estimate the pre-miRNA content and to predict the location of precursor miRNAs in genomic sequences. This method can be used to guide experiments to find both miRNAs that are evolutionarily conserved as well as species-specific miRNAs such as those known now to exist in viruses. Here we applied our method to the discovery of clustered, probably co-transcribed, miRNAs in human, mouse and rat. We have shown that our method successfully identifies evolutionarily conserved miRNAs that have been missed by various other methods that are based on cross-species comparisons. Most of the novel miRNAs that we discovered reside in a conserved imprinted locus from chromosome 14 in human, 12 in mouse and 6 in rat and on chromosome X (in all species). In these regions we found species-specific patterns of miRNA duplication and diversification. The web interface to our prediction method can be accessed on the world-wide-web [28].

## Methods

### Extraction of genomic regions with robust secondary structures

We determine genomic regions with context-independent stem loop secondary structures (shortly "robust" secondary structures) as follows. We move a window of length *L *across the entire input RNA sequence in a stepwise manner, shifting by an amount Δ*L *at each step. For each window position we compute the minimal free-energy secondary structure of the corresponding sequence using the *RNAfold *program of the Vienna package [29]. We store the nucleotide pairs of this structure into a table with all the pairs that occurred in at least one structure, and at the end, we determine the preservation rate ("robustness") *r *for every nucleotide pair (*i*, *j*) in the table. This is defined as:

r=number of windows containing the nucleotide pair (i,j)number of windows containing both nucleotides i and j.     (1)
 MathType@MTEF@5@5@+=feaafiart1ev1aaatCvAUfKttLearuWrP9MDH5MBPbIqV92AaeXatLxBI9gBaebbnrfifHhDYfgasaacH8akY=wiFfYdH8Gipec8Eeeu0xXdbba9frFj0=OqFfea0dXdd9vqai=hGuQ8kuc9pgc9s8qqaq=dirpe0xb9q8qiLsFr0=vr0=vr0dc8meaabaqaciGacaGaaeqabaqabeGadaaakeaacqWGYbGCcqGH9aqpdaWcaaqaaiabb6gaUjabbwha1jabb2gaTjabbkgaIjabbwgaLjabbkhaYjabbccaGiabb+gaVjabbAgaMjabbccaGiabbEha3jabbMgaPjabb6gaUjabbsgaKjabb+gaVjabbEha3jabbohaZjabbccaGiabbogaJjabb+gaVjabb6gaUjabbsha0jabbggaHjabbMgaPjabb6gaUjabbMgaPjabb6gaUjabbEgaNjabbccaGiabbsha0jabbIgaOjabbwgaLjabbccaGiabb6gaUjabbwha1jabbogaJjabbYgaSjabbwgaLjabb+gaVjabbsha0jabbMgaPjabbsgaKjabbwgaLjabbccaGiabbchaWjabbggaHjabbMgaPjabbkhaYjabbccaGiabbIcaOiabdMgaPjabcYcaSiabdQgaQjabcMcaPaqaaiabb6gaUjabbwha1jabb2gaTjabbkgaIjabbwgaLjabbkhaYjabbccaGiabb+gaVjabbAgaMjabbccaGiabbEha3jabbMgaPjabb6gaUjabbsgaKjabb+gaVjabbEha3jabbohaZjabbccaGiabbogaJjabb+gaVjabb6gaUjabbsha0jabbggaHjabbMgaPjabb6gaUjabbMgaPjabb6gaUjabbEgaNjabbccaGiabbkgaIjabb+gaVjabbsha0jabbIgaOjabbccaGiabb6gaUjabbwha1jabbogaJjabbYgaSjabbwgaLjabb+gaVjabbsha0jabbMgaPjabbsgaKjabbwgaLjabbohaZjabbccaGiabdMgaPjabbccaGiabbggaHjabb6gaUjabbsgaKjabbccaGiabdQgaQbaacqGGUaGlcaWLjaGaaCzcamaabmaabaGaeGymaedacaGLOaGaayzkaaaaaa@BBCB@

Choosing a minimal robustness value *r*_min_, we reconstruct the secondary structure elements (including stem loops) that occur with rate *r *≥ *r*_min _using the following property of the nearest-neighbor energy model used in the the secondary structure calculation [30]. Given two different sequences containing both the subsequence corresponding to the interval [*i *- 1, *j *+ 1], if the pair (*i*, *j*) is present in the minimal free-energy structures of the two sequences, then the secondary structure pairing pattern of the subsequence [*i*, *j*] is exactly the same for the two sequences. Therefore, if a pair (*i*, *j*) appears with a robustness *r *in the table, then all pairs in the table belonging to the common minimal free energy structure of the subsequence [*i *+ 1, *j *- 1] in overlapping windows will appear with a robustness of at least *r*. We use this property to reconstruct the secondary structure elements preserved with a minimal rate of *r*_min_. In our implementation we take *r*_min _= 0.9, and keep only the genomic regions where a single stem of at least 15 nucleotides is present in the robust secondary structure. Finally, we fix the windowing parameters *L *and Δ*L*. *L *should correspond to the length of known miRNA precursors, which can vary between hundreds and thousands of nucleotides for the primary transcripts and between 50 and 70 nucleotides for pre-miRNAs. The constraint on Δ*L *is set such as to allow us to collect sufficient data to make a statistically meaningful estimation of structure robustness. To fulfill all these requirements while keeping the calculation time within reasonable limits, we use two combinations of (*L*,Δ*L*) values, namely (500,25) and (1000,50), and then take the intersection of the sets of predicted regions with robust secondary structures.

Application of the above procedure to genomic regions of 20 kb around the Rfam6.0 miRNA loci [18] shows that 89%/88%/89% (203/205/166 out of 227/232/186) of the known precursors of human/mouse/rat miRNAs overlap with a robust stem loop. This cross-check shows that the secondary structure "robustness" criteria is very appropriate to make a first selection of candidate miRNA precursor stem loops.

### Classification of stem loops using a support vector machine

In order to construct a support vector machine (SVM) classifier for the candidate stem loops, we need a set positive and a set of negative examples of miRNA precursors. As positive examples, we use the complete set of human pre-miRNAs in the Rfam repository. As negative examples we use random subsequences isolated from tRNA, rRNA and mRNA genes. In cases where the input sequence was too short to enable us to perform the robust stem identification as described above (e.g. in the case of the short tRNA genes), we pad the input sequence with random sequence using the mono-nucleotide frequencies of the input sequence. To get a good sampling of the space of sequences that the transcription and miRNA processing machinery may encounter in the cell, we add to the set of negative examples genomic regions isolated from random positions in the human genome, as well as the genomic regions from various viruses. Given that the fraction of genome encoding miRNA genes is quite small, it is highly unlikely that the sequences that we have chosen this way contain miRNA precursors. We have a total of 178 positive examples (i.e. the human precursor miRNAs from Rfam4.0) and 5395 negative examples. Thus the fraction of positives is the training set is of the order of a few percent, similar to what we think is the case in the human genome. Analyzing the known examples [18], we and others find that miRNA precursors generally assume simple hairpin structures (except some member of the let-7 family), longer than about 50 nucleotides. The fraction of paired nucleotides in the stem is relatively high, and the internal loops, if present, tend to be symmetrical. The hairpin loop appears to be relatively short, of at most 20 nucleotides. The nucleotide composition in the stem is generally balanced, as is the number of A-U, G-C and G-U pairs. Based on such considerations we use the *RNAfold *program to calculate the minimal free energy secondary structure [29] and then we describe each stem loop (positive/negative example or candidate pre-miRNA) in terms of the following sets of features:

1. Statistics computed over the entire hairpin structure: 16 features given in Table 2;

2. Statistics computed over the longest symmetrical region of the stem, i.e. the longest region without any asymmetrical loops: 10 features given in Table 3;

3. Statistics computed over the longest region in which the difference between the 5' and 3' components of asymmetrical loops is not larger than Δ*l *(this region is called "relaxed symmetry region"): 11 features given in Table 4.

4. Statistics computed over all windows of length corresponding to mature miRNA length *l*_*m *_that we could place on the candidate stem loop, in order to zoom onto the region of the mature miRNAs: 3 features given in Table 5.

Note that there are only two parameters in these computations: the maximally allowed asymmetry Δ*l *in a region with "relaxed symmetry", and the assumed length of the mature miRNA *l*_*m*_. We choose these parameters so as to minimize the number of misclassified examples in our training set. This minimum occurs at the values 4 and 20, respectively.

**Figure 4:**
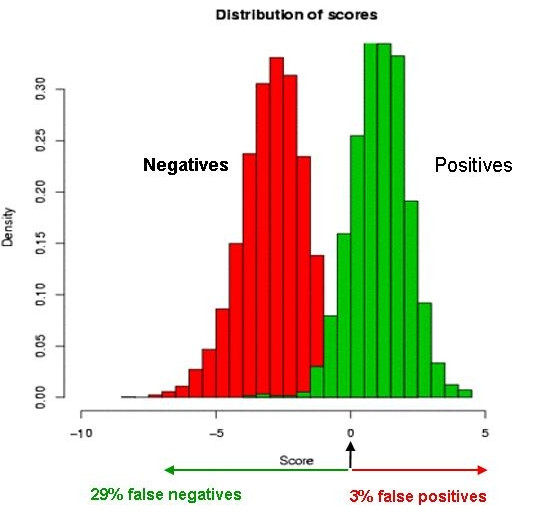
**SVM training set score distributions**. Normalized distributions of the SVM scores for the positive and negative stem loop examples used for the SVM training. The good separation between the two sets indicates that the SVM performs well in recognizing the miRNA precursor specific features.

For the SVM, we use the "SVMlight" implementation of Joachims and a quadratic kernel [31]. This implementation allows us to choose an additional cost for penalizing misclassified positive relative to negative examples. We chose this value such as to get a reasonable recovery rate of known pre-miRNAs, while keeping a low false positive rate. The distributions of scores of the training sets are shown in Figure 4. In short, the model that we have constructed recovers 71% of the positive pre-miRNA examples with robust stems, with a false positive rate of 3%. The features to which the SVM has assigned the largest positive weights are the stem length, the length of the longest symmetrical region, number of A-U and number of G-C base pairs in the "relaxed symmetry" region. The features with the largest negative weights are the free energy of folding, the number of nucleotides in symmetrical and asymmetrical loops in the "relaxed symmetry" region, and the average size of asymmetrical loops. These conform to prior knowledge [13].

### Estimation of the pre-miRNA content of a sequence

The number of pre-miRNA predictions obtained from the SVM classifier depends on the value of the score threshold, which is somewhat arbitrary. We show here that our approach can provide an estimate of the pre-miRNA content of a sequence which is independent of this threshold.

According to the assumptions made previously, the features that we use to decide whether a candidate stem loop is a miRNA precursor are contained in RNA sequence and secondary structures only, and are combined into a score using the SVM model. If this assumption holds, then the probability *p *that a robust stem loop contains a miRNA is a function of the score *s *only, and we can compute the overall pre-miRNA content of a sequence based on the distributions of scores for the set of positives (*S*^+^), the set of negatives (*S*^-^), and the set of candidate stems in a given genomic sequence (*S*_*C*_). We expect that *p*(*s*) has a step-like monotonic behavior, being close to 0 for large negative values of *s *and becoming asymptotically close to 1 for large positive scores. In order to concretely specify the function *p*(*s*), we first choose a suitable parametric functional expression such as

p(s)=12(1+tanh (as+b))     (2)
 MathType@MTEF@5@5@+=feaafiart1ev1aaatCvAUfKttLearuWrP9MDH5MBPbIqV92AaeXatLxBI9gBaebbnrfifHhDYfgasaacH8akY=wiFfYdH8Gipec8Eeeu0xXdbba9frFj0=OqFfea0dXdd9vqai=hGuQ8kuc9pgc9s8qqaq=dirpe0xb9q8qiLsFr0=vr0=vr0dc8meaabaqaciGacaGaaeqabaqabeGadaaakeaacqWGWbaCcqGGOaakcqWGZbWCcqGGPaqkcqGH9aqpdaWcaaqaaiabigdaXaqaaiabikdaYaaacqGGOaakcqaIXaqmcqGHRaWkcqqG0baDcqqGHbqycqqGUbGBcqqGObaAcqqGGaaicqqGOaakcqWGHbqycqWGZbWCcqGHRaWkcqWGIbGycqGGPaqkcqGGPaqkcaWLjaGaaCzcamaabmaabaGaeGOmaidacaGLOaGaayzkaaaaaa@4846@

and then fix its parameters *a *and *b *by maximizing the likelihood of the training set, defined as

L(a,b)=∏i∈S+p(si)∏j∈S−p(sj).     (3)
 MathType@MTEF@5@5@+=feaafiart1ev1aaatCvAUfKttLearuWrP9MDH5MBPbIqV92AaeXatLxBI9gBaebbnrfifHhDYfgasaacH8akY=wiFfYdH8Gipec8Eeeu0xXdbba9frFj0=OqFfea0dXdd9vqai=hGuQ8kuc9pgc9s8qqaq=dirpe0xb9q8qiLsFr0=vr0=vr0dc8meaabaqaciGacaGaaeqabaqabeGadaaakeaacqWGmbatcqGGOaakcqWGHbqycqGGSaalcqWGIbGycqGGPaqkcqGH9aqpdaqeqbqaaiabdchaWjabcIcaOiabdohaZnaaBaaaleaacqWGPbqAaeqaaOGaeiykaKcaleaacqWGPbqAcqGHiiIZcqWGtbWudaahaaadbeqaaiabgUcaRaaaaSqab0Gaey4dIunakmaarafabaGaemiCaaNaeiikaGIaem4Cam3aaSbaaSqaaiabdQgaQbqabaGccqGGPaqkcqGGUaGlcaWLjaGaaCzcamaabmaabaGaeG4mamdacaGLOaGaayzkaaaaleaacqWGQbGAcqGHiiIZcqWGtbWudaahaaadbeqaaiabgkHiTaaaaSqab0Gaey4dIunaaaa@5369@

If we now make the assumption that all candidates *i *∊ *S*_*C *_are independent from another, the number of miRNA precursors *E *is given by a sum of independent Bernoulli distributions, each of them being characterized by its own probability 0 ≤ *p*(*s*_*i*_) ≤ 1. As a consequence, the expected value of *E *and its error Δ*E *are given by

E±ΔE=∑i∈SCp(si)±∑i∈SCp(si)(1−p(si)).     (4)
 MathType@MTEF@5@5@+=feaafiart1ev1aaatCvAUfKttLearuWrP9MDH5MBPbIqV92AaeXatLxBI9gBaebbnrfifHhDYfgasaacH8akY=wiFfYdH8Gipec8Eeeu0xXdbba9frFj0=OqFfea0dXdd9vqai=hGuQ8kuc9pgc9s8qqaq=dirpe0xb9q8qiLsFr0=vr0=vr0dc8meaabaqaciGacaGaaeqabaqabeGadaaakeaacqWGfbqrcqGHXcqScqqHuoarcqWGfbqrcqGH9aqpdaaeqbqaaiabdchaWjabcIcaOiabdohaZnaaBaaaleaacqWGPbqAaeqaaOGaeiykaKIaeyySae7aaOaaaeaadaaeqbqaaiabdchaWjabcIcaOiabdohaZnaaBaaaleaacqWGPbqAaeqaaOGaeiykaKIaeiikaGIaeGymaeJaeyOeI0IaemiCaaNaeiikaGIaem4Cam3aaSbaaSqaaiabdMgaPbqabaGccqGGPaqkcqGGPaqkaSqaaiabdMgaPjabgIGiolabdofatnaaBaaameaacqWGdbWqaeqaaaWcbeqdcqGHris5aaWcbeaakiabc6caUiaaxMaacaWLjaWaaeWaaeaacqaI0aanaiaawIcacaGLPaaaaSqaaiabdMgaPjabgIGiolabdofatnaaBaaameaacqWGdbWqaeqaaaWcbeqdcqGHris5aaaa@5EB8@

We can also calculate the probability that a given genome encodes any given integer number *m *of miRNAs precursors. Defining a generating function as

G(z)=∏i∈SC(1−p(si)+zp(si))     (5)
 MathType@MTEF@5@5@+=feaafiart1ev1aaatCvAUfKttLearuWrP9MDH5MBPbIqV92AaeXatLxBI9gBaebbnrfifHhDYfgasaacH8akY=wiFfYdH8Gipec8Eeeu0xXdbba9frFj0=OqFfea0dXdd9vqai=hGuQ8kuc9pgc9s8qqaq=dirpe0xb9q8qiLsFr0=vr0=vr0dc8meaabaqaciGacaGaaeqabaqabeGadaaakeaacqWGhbWrcqGGOaakcqWG6bGEcqGGPaqkcqGH9aqpdaqeqbqaaiabcIcaOiabigdaXiabgkHiTiabdchaWjabcIcaOiabdohaZnaaBaaaleaacqWGPbqAaeqaaOGaeiykaKIaey4kaSIaemOEaONaemiCaaNaeiikaGIaem4Cam3aaSbaaSqaaiabdMgaPbqabaGccqGGPaqkcqGGPaqkcaWLjaGaaCzcamaabmaabaGaeGynaudacaGLOaGaayzkaaaaleaacqWGPbqAcqGHiiIZcqWGtbWudaWgaaadbaGaem4qameabeaaaSqab0Gaey4dIunaaaa@4F4A@

the expression for the probability of having exactly *m *miRNAs is found to be

P(m)=1m!∂m∂zmG(z)|z=0     (6)=∑m−uplesj1,…,jm∈SCp(sj1)…p(sjm)∏j∉{j1,…,jm}(1−p(sj))
 MathType@MTEF@5@5@+=feaafiart1ev1aaatCvAUfKttLearuWrP9MDH5MBPbIqV92AaeXatLxBI9gBaebbnrfifHhDYfgasaacH8akY=wiFfYdH8Gipec8Eeeu0xXdbba9frFj0=OqFfea0dXdd9vqai=hGuQ8kuc9pgc9s8qqaq=dirpe0xb9q8qiLsFr0=vr0=vr0dc8meaabaqaciGacaGaaeqabaqabeGadaaakqaaeeqaaiabdcfaqjabcIcaOiabd2gaTjabcMcaPiabg2da9maalaaabaGaeGymaedabaGaemyBa0MaeiyiaecaamaalaaabaGaeyOaIy7aaWbaaSqabeaacqWGTbqBaaaakeaacqGHciITcqWG6bGEdaahaaWcbeqaaiabd2gaTbaaaaGccqWGhbWrcqGGOaakcqWG6bGEcqGGPaqkdaabbaqaaiabdQha6jabg2da9iabicdaWaGaay5bSdGaaCzcaiaaxMaacaWLjaGaaCzcaiaaxMaacaWLjaGaaCzcamaabmaabaGaeGOnaydacaGLOaGaayzkaaaabaGaeyypa0ZaaabuaeaacqWGWbaCdaqadaqaaiabdohaZnaaBaaaleaacqWGQbGAdaWgaaadbaGaeGymaedabeaaaSqabaaakiaawIcacaGLPaaacqWIMaYscqWGWbaCdaqadaqaaiabdohaZnaaBaaaleaacqWGQbGAdaWgaaadbaGaemyBa0gabeaaaSqabaaakiaawIcacaGLPaaaaSqaaiabd2gaTjabgkHiTiabbwha1jabbchaWjabbYgaSjabbwgaLjabbohaZnaaBaaameaacqWGQbGAdaWgaaqaaiabigdaXaqabaGaeiilaWIaeSOjGSKaeiilaWIaemOAaO2aaSbaaeaacqWGTbqBaeqaaiabgIGiolabdofatnaaBaaabaGaem4qameabeaaaeqaaaWcbeqdcqGHris5aOWaaebuaeaacqGGOaakcqaIXaqmcqGHsislcqWGWbaCcqGGOaakcqWGZbWCdaWgaaWcbaGaemOAaOgabeaakiabcMcaPiabcMcaPaWcbaGaemOAaOMaeyycI8Saei4EaSNaemOAaO2aaSbaaWqaaiabigdaXaqabaWccqGGSaalcqWIMaYscqGGSaalcqWGQbGAdaWgaaadbaGaemyBa0gabeaaliabc2ha9bqab0Gaey4dIunaaaaa@8F83@

The quantiles of the distribution *P*(*m*) are used to estimate the minimal number of expected miRNA precursors. Define the quantity

**Figure 5:**
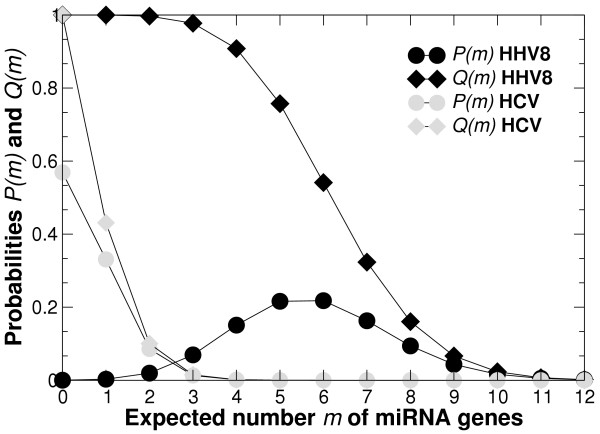
**Estimation of the pre-miRNA content for HHV8 and HCV**. Plot of the probability *P*(*m*) of a given virus to encode exactly *m *pre-miRNAs (Equation 6) and probability *Q*(*m*) to encode at least *m *pre-miRNAs (Equation 7). Equation 4 allows to calculate the corresponding number of expected pre-miRNAs, 5.8 ± 1.8 for HHV8 and 0.5 ± 0.7 for HCV. These values correspond to the mean and standard deviation of the distribution *P*(*m*) shown on the figure.

Q(m)=1−∑n<mP(n)=∑n≥mP(n),     (7)
 MathType@MTEF@5@5@+=feaafiart1ev1aaatCvAUfKttLearuWrP9MDH5MBPbIqV92AaeXatLxBI9gBaebbnrfifHhDYfgasaacH8akY=wiFfYdH8Gipec8Eeeu0xXdbba9frFj0=OqFfea0dXdd9vqai=hGuQ8kuc9pgc9s8qqaq=dirpe0xb9q8qiLsFr0=vr0=vr0dc8meaabaqaciGacaGaaeqabaqabeGadaaakeaacqWGrbqucqGGOaakcqWGTbqBcqGGPaqkcqGH9aqpcqaIXaqmcqGHsisldaaeqbqaaiabdcfaqjabcIcaOiabd6gaUjabcMcaPiabg2da9maaqafabaGaemiuaaLaeiikaGIaemOBa4MaeiykaKIaeiilaWIaaCzcaiaaxMaadaqadaqaaiabiEda3aGaayjkaiaawMcaaaWcbaGaemOBa4MaeyyzImRaemyBa0gabeqdcqGHris5aaWcbaGaemOBa4MaeyipaWJaemyBa0gabeqdcqGHris5aaaa@4EA0@

then if *n *is the largest integer such that *Q*(*n*) ≥ 0.99, then *n *is the number of pre-miRNAs that we estimate to be encoded in the considered genome with 99% confidence. Figure 5 shows an illustration of these distributions for two viruses [17].

We perform a cross-check for the above approach by applying it to the full training set of the SVM, merging the positive and negative stems loop into one single set. The expected number of pre-miRNAs from Equation 4 is 157.2 ± 6.3. The number of pre-miRNAs present with 99% confidence in the training set is 143. These numbers are in total agreement with the sensitivity of the SVM, according to which 0.7 = 155 of the positives should be correctly classified.

## Authors' contributions

AS and MZ performed the bioinformatic work. NP helped in setting up the web server. PL, AA, SP, MJB, and TT provided the cloning data. EvN developed the probabilistic pre-miRNA content estimation method. MZ supervised the collaboration between the various people.

## Supplementary Material

Additional File 1 **All predictions, human**. The table contains exhaustive information about all the predicted miRNA precursors that have been assumed to co-transcribed with a known human miRNA present in the Rfam6.0 set. The latter may be the one given in the column "closest miRNA", characterized by the smallest genomic distance to the prediction and a common transcription sense. A row has a color background when it contains a prediction that has been confirmed by cloning. The genomic coordinates are given for the hg17 human genome assembly. In the columns about cloning and cross-species conservation the letters "h", "m", and "r" mean the corresponding property being satisfied for human, mouse, and rat, respectively. The last column show (not yet confirmed) pre-miRNA predictions obtained using methods based on cross-species conservation. The first letters "B", "L" and "X" indicate results by Berezikov *et al. *[10], Legendre *et al. *[15], and Xie *et al. *[21], respectively. The other letters are the identifiers given by these authors to the corresponding predictions. We also indicate the recent predictions by Altuvia *et al.*, labeled by an "A", who studied human miRNA clusters using a different approach [23]. Click here for file

Additional File 2 **All predictions, mouse**. The same as the Additional file 1, but for the mouse predictions. The genome assembly use for the coordinates is mm5. Click here for file

Additional File 3 **All predictions, rat**. The same as the Additional file 1, but for the rat predictions. The genome assembly used for the coordinates is rn3. Click here for file

Additional File 4 **All confirmed, human**. Details of the confirmed miRNAs for human. The cloning frequency indicates the number of distinct small RNA cloned sequences found in the comprehensive cloning set that match our predicted pre-miRNA, "h", "m", and "r" corresponding to human, mouse, and rat, respectively. The secondary structure uses the text display from Mfold [32]. The last column gives the best homologs (within at most 5 mismatches) found in our confirmed predictions, in the false negative sets (Additional files 7, 8, and 9) and in Rfam7.1 (latest release at the time of publication). Notice MP-61 with good cloning evidence but an unusual position in the secondary structure. Click here for file

Additional File 5 **All confirmed, mouse**. Same as the Additional file 4, but for mouse. Notice that the case MP-28 is identical to MP-61. Click here for file

Additional File 6 **All confirmed, rat**. Same as the Additional file 4, but for rat. RP-66 is identical to HP-61 and MP-28. Click here for file

Additional File 7 **False negatives, human**. Set of false negatives for human, i.e. stem loop candidates with a negative SVM score but which have a cloning evidence. The layout is identical to the table from the Additional files 4, 5, and 6. The Additional column "Other predictions" is filled as in the additional files 1, 2, and 3. Click here for file

Additional File 8 **False negatives, mouse**. Set of false negatives for mouse, similar to the Additional file 7. Click here for file

Additional File 9 **False negative, rat**. Set of false negatives for rat, similar to the Additional file 7. Notice the unusual position of RN-4 in the secondary structure. Click here for file

Additional File 10 Additional files 1, 2, 3, 4, 5, 6, 7, 8, 9**in text format**. This archive contains the text file versions of the Additional files 1, 2, 3, 4, 5, 6, 7, 8, 9 as TAB-separated lists. They contain additionally the explicit genomic sequences of all the predictions. Click here for file
