# Supplementary material for: Identification of clustered microRNAs using an ab initio prediction method
Source: BMC Bioinformatics. 2005 Nov 7;6:267. doi: 10.1186/1471-2105-6-267 (PMC1315341; doi:10.1186/1471-2105-6-267)
Supplement: Additional File 1 — All predictions, human. The table contains exhaustive information about all the predicted miRNA precursors that have been assumed to co-transcribed with a known human miRNA present in the Rfam6.0 set. The latter may be the one given in the column "closest miRNA", characterized by the smallest genomic distance to the prediction and a common transcription sense. A row has a color background when it contains a prediction that has been confirmed by cloning. The genomic coordinates are given for the hg17 human genome assembly. In the columns about cloning and cross-species conservation the letters "h", "m", and "r" mean the corresponding property being satisfied for human, mouse, and rat, respectively. The last column show (not yet confirmed) pre-miRNA predictions obtained using methods based on cross-species conservation. The first letters "B", "L" and "X" indicate results by Berezikov et al. [10], Legendre et al. [15], and Xie et al. [21], respectively. The other letters are the identifiers given by these authors to the corresponding predictions. We also indicate the recent predictions by Altuvia et al., labeled by an "A", who studied human miRNA clusters using a different approach [23]. [file 1471-2105-6-267-S1.pdf]

Name	Chromosome	Genomic coordinates	SVM score	Cloning	Conservation			Closest miRNA	Other predictions
HP-1	chr1	40887341-40887436,+	3.02	-	-	-	-	hsa-mir-30e	
HP-2	chr1	40898684-40898762,+	0.44	-	-	-	-	hsa-mir-30c-1	
HP-3	chr1	65232147-65232234,-	2.72	-	-	-	-	hsa-mir-101-1	
HP-4	chr1	98222840-98222917,-	0.48	-	m|r	m|r	r	hsa-mir-137	B648
HP-5	chr1	153211595-153211689,-	0.95	-	-	-	-	hsa-mir-9-1	
HP-6	chr2	136262939-136263027,+	0.06	-	-	-	-	hsa-mir-128a	
HP-7	chr2	176831714-176831785,+	0.04	-	-	-	-	hsa-mir-10b	
HP-8	chr3	37982219-37982304,+	0.46	-	-	-	-	hsa-mir-26a-1	
HP-9	chr3	161610634-161610741,+	0.10	-	-	-	-	hsa-mir-16-2	
HP-10	chr5	54503853-54503934,-	2.09	-	-	-	-	hsa-mir-449	
HP-11	chr5	148785556-148785635,+	1.21	-	-	-	-	hsa-mir-143	
HP-12	chr5	179377228-179377312,-	0.11	-	-	-	-	hsa-mir-340	
HP-13	chr6	52127783-52127846,+	0.44	-	-	-	-	hsa-mir-133b	
HP-14	chr6	72141346-72141459,-	0.74	-	m|r	r	-	hsa-mir-30c-2	
HP-15	chr7	129017934-129018046,-	4.42	-	m	m	-	hsa-mir-183	
HP-16	chr7	129722221-129722300,+	0.58	-	m	-	-	hsa-mir-335	
HP-17	chr8	141804157-141804256,-	2.00	-	-	-	-	hsa-mir-151	
HP-18	chr8	141813107-141813217,-	0.09	-	-	-	-	hsa-mir-151	
HP-19	chr9	120085235-120085329,-	0.50	-	-	-	-	hsa-mir-147	
HP-20	chr11	553994-554064,-	0.31	-	-	-	-	hsa-mir-210	
HP-21	chr11	559747-559827,-	0.82	-	-	-	-	hsa-mir-210	
HP-22	chr11	74715663-74715760,-	1.78	-	m	m	-	hsa-mir-326	
HP-23	chr11	74716123-74716211,-	2.05	-	-	-	-	hsa-mir-326	
HP-24	chr11	74716486-74716607,-	0.44	-	m|r	-	-	hsa-mir-326	
HP-25	chr11	121485215-121485296,-	2.41	-	-	-	-	hsa-mir-125b-1	
HP-26	chr11	121528013-121528085,-	0.47	-	-	-	-	hsa-mir-100	
HP-27	chr12	52668365-52668434,+	0.85	-	-	-	-	hsa-mir-196a-2	
HP-28	chr13	49519429-49519510,-	0.81	-	-	-	-	hsa-mir-16-1	
HP-29	chr14	99636013-99636131,+	0.85	-	-	-	-	hsa-mir-342	
HP-30	chr14	100405160-100405230,+	0.12	h	m|r	m|r	-	hsa-mir-337	B202, X52
HP-31	chr14	100420573-100420666,+	1.27	h	m	m	-	hsa-mir-136	
HP-32	chr14	100447566-100447644,+	0.42	-	-	-	-	hsa-mir-370	
HP-33	chr14	100562875-100562954,+	1.29	h	m|r	m|r	-	hsa-mir-323	ANMH46
HP-34	chr14	100563199-100563264,+	0.25	h	-	-	-	hsa-mir-323	
HP-35	chr14	100565724-100565804,+	1.40	h	m|r	m|r	r	hsa-mir-323	B209, X61
HP-36	chr14	100569845-100569926,+	1.67	-	m|r	m|r	r	hsa-mir-368	X124
HP-37	chr14	100576159-100576238,+	1.31	h	m|r	m|r	m|r	hsa-mir-368	
HP-38	chr14	100576535-100576620,+	0.98	-	m|r	m|r	m|r	hsa-mir-376a	ANMH47, X205
HP-39	chr14	100580290-100580371,+	0.93	-	-	-	-	hsa-mir-381	
HP-40	chr14	100582545-100582628,+	1.28	h	m|r	m|r	-	hsa-mir-381	B212, X199
HP-41	chr14	100583411-100583488,+	1.17	h	m|r	m|r	m	hsa-mir-381	B213, X177
HP-42	chr14	100584748-100584838,+	0.30	h	m|r	m|r	m|r	hsa-mir-381	B214
HP-43	chr14	100585651-100585730,+	1.10	-	-	-	-	hsa-mir-381	
HP-44	chr14	100588536-100588615,+	1.11	h	m|r	m|r	m	hsa-mir-382	
HP-45	chr14	100591505-100591585,+	1.41	m	m|r	m|r	m	hsa-mir-134	ANMH51, B217, X23
HP-46	chr14	100596673-100596749,+	1.34	-	m|r	m|r	m|r	hsa-mir-154	ANMH53, B219, X161
HP-47	chr14	100601390-100601468,+	0.74	h|m	m|r	m|r	-	hsa-mir-369	ANMH55, B221
HP-48	chr14	100601548-100601614,+	0.18	m	m|r	m|r	-	hsa-mir-369	ANMH56
HP-49	chr14	100602002-100602081,+	0.78	h|m	m|r	m|r	m|r	hsa-mir-369	ANMH57, B565, X26
HP-50	chr15	29148489-29148618,-	1.15	-	m	-	-	hsa-mir-211	
HP-51	chr15	61945887-61945958,-	0.69	-	-	-	-	hsa-mir-422a	
HP-52	chr15	61956585-61956672,-	1.23	-	-	-	-	hsa-mir-422a	
HP-53	chr15	77283739-77283834,+	2.79	-	-	-	-	hsa-mir-184	
HP-54	chr15	77286586-77286662,+	0.19	-	-	-	-	hsa-mir-184	
HP-55	chr15	86952348-86952421,+	0.43	-	r	r	-	hsa-mir-7-2	
HP-56	chr16	14305325-14305407,+	1.25	h	m	m	m	hsa-mir-365-1	B707
HP-57	chr16	55455432-55455558,+	8.08	-	m	m	-	hsa-mir-138-2	
HP-58	chr16	65802725-65802850,-	1.03	-	r	-	-	hsa-mir-328	
HP-59	chr17	1557905-1558005,-	2.40	-	r	r	-	hsa-mir-22	
HP-60	chr17	7077268-7077349,-	0.04	-	-	-	-	hsa-mir-324	
HP-61	chr17	24212514-24212583,-	0.83	h|m	m|r	m|r	m|r	hsa-mir-144	ANMH67, B919, X65

HP-62	chr17	26925895-26925998,+	0.15	-	-	-	-	hsa-mir-365-2	
HP-63	chr17	54574700-54574820,-	4.57	-	-	-	-	hsa-mir-301	
HP-64	chr17	54583611-54583698,-	1.96	-	m	-	-	hsa-mir-301	
HP-65	chr17	55276486-55276565,+	1.08	-	-	-	-	hsa-mir-21	
HP-66	chr17	76710147-76710253,-	0.16	-	-	-	-	hsa-mir-338	
HP-67	chr18	54262074-54262128,+	0.20	-	-	-	-	hsa-mir-122a	
HP-68	chr19	10794434-10794541,-	4.81	-	m|r	m	-	hsa-mir-199a-1	
HP-69	chr19	50828807-50828889,-	1.45	-	-	-	-	hsa-mir-330	
HP-70	chr19	58987053-58987142,+	0.42	-	-	-	-	hsa-mir-373	
HP-71	chr19	58991703-58991827,+	4.12	-	m|r	m|r	m	hsa-mir-373	
HP-72	chr20	61288007-61288074,+	0.01	-	-	-	-	hsa-mir-124a-3	
HP-73	chr21	16826662-16826746,+	0.07	-	-	-	-	hsa-mir-99a	
HP-74	chr21	25875807-25875895,+	0.52	-	-	-	-	hsa-mir-155	
HP-75	chr22	18387174-18387296,+	5.67	-	m	m	-	hsa-mir-185	
HP-76	chrX	49466772-49466857,+	0.09	-	-	-	-	hsa-mir-188	
HP-77	chrX	49476605-49476676,+	0.32	h|m	m|r	m|r	-	hsa-mir-188	B121, X144
HP-78	chrX	49477366-49477449,+	0.20	-	m|r	m|r	m	hsa-mir-188	B122
HP-79	chrX	65017860-65017962,+	0.81	-	-	-	-	hsa-mir-223	
HP-80	chrX	73289960-73290065,-	2.01	h	m|r	-	-	hsa-mir-374	
HP-81	chrX	84968305-84968369,-	0.14	-	-	-	-	hsa-mir-361	
HP-82	chrX	113872348-113872431,+	0.28	-	m|r	m	-	hsa-mir-448	
HP-83	chrX	122415494-122415578,-	0.39	-	-	-	-	hsa-mir-220	
HP-84	chrX	133019128-133019229,-	1.95	-	-	-	-	hsa-mir-92-2	
HP-85	chrX	133028921-133029009,-	1.14	h|m	m|r	m|r	-	hsa-mir-92-2	ANMH3, B343, X211
HP-86	chrX	133400068-133400148,-	1.59	h	m|r	m|r	-	hsa-mir-450	X138
HP-87	chrX	133405878-133405948,-	0.80	h	m|r	m|r	-	hsa-mir-424	B973, X141
HP-88	chrX	150798672-150798748,-	1.02	h	m	m	-	hsa-mir-224	AMH1, X154
HP-89	chrX	151232472-151232559,-	2.56	-	m|r	m	-	hsa-mir-105-2	
