# Supplementary material for: Identification of clustered microRNAs using an ab initio prediction method
Source: BMC Bioinformatics. 2005 Nov 7;6:267. doi: 10.1186/1471-2105-6-267 (PMC1315341; doi:10.1186/1471-2105-6-267)
Supplement: Additional File 2 — All predictions, mouse. The same as the Additional file 1, but for the mouse predictions. The genome assembly use for the coordinates is mm5. [file 1471-2105-6-267-S2.pdf]

Name	Chromosome	Genomic coordinates	SVM score	Cloning	Conservation			Closest miRNA	Other predictions
MP-1	chr1	132070573-132070662,+	0.15	-	r	r	-	mmu-mir-135b	
MP-2	chr1	194939223-194939296,+	0.97	-	r	r	-	mmu-mir-29c	
MP-3	chr2	38822522-38822594,+	0.05	-	-	-	-	mmu-mir-181b-2	
MP-4	chr3	88712241-88712321,+	0.5	-	h|r	h|r	-	mmu-mir-9-1	
MP-5	chr3	128025558-128025632,+	0.78	h|m	h	h	-	mmu-mir-302	
MP-6	chr3	158156790-158156878,+	0.53	-	r	-	-	mmu-mir-186	
MP-7	chr4	56172443-56172536,-	0.01	-	r	r	-	mmu-mir-32	
MP-8	chr4	99698758-99698875,-	0.16	-	h|r	h|r	h|r	mmu-mir-101a	
MP-9	chr5	135550619-135550746,-	0.34	-	r	-	-	mmu-mir-106b	
MP-10	chr6	30049436-30049516,-	0.11	-	-	-	-	mmu-mir-182	
MP-11	chr6	30053316-30053400,-	0.6	-	r	r	-	mmu-mir-96	
MP-12	chr6	30937279-30937355,-	0.99	-	-	-	-	mmu-mir-29a	
MP-13	chr6	30948760-30948857,-	0.5	-	-	-	-	mmu-mir-29b-1	
MP-14	chr6	51213052-51213135,-	0.06	-	-	-	-	mmu-mir-148a	
MP-15	chr6	125337858-125337931,-	0.42	-	-	-	-	mmu-mir-200c	
MP-16	chr7	32507703-32507775,+	0.08	-	-	-	-	mmu-mir-150	
MP-17	chr7	51204065-51204177,+	2.15	-	h|r	r	-	mmu-mir-211	
MP-18	chr7	88830215-88830288,+	0.31	-	r	r	-	mmu-mir-139	
MP-19	chr7	127610625-127610714,-	0.62	-	r	r	-	mmu-mir-202	
MP-20	chr8	83456359-83456470,-	2.76	-	h|r	r	-	mmu-mir-181c	
MP-21	chr8	83472287-83472363,+	0.15	-	r	r	-	mmu-mir-23a	
MP-22	chr8	83488943-83489033,+	1.76	-	h|r	h	-	mmu-mir-24-2	
MP-23	chr9	106115667-106115762,+	0.11	-	-	-	-	mmu-let-7g	
MP-24	chr10	127120840-127120960,+	0.9	m	r	-	-	mmu-mir-26a-2	
MP-25	chr11	43005924-43005996,-	0.69	-	r	r	-	mmu-mir-146	
MP-26	chr11	49702031-49702111,+	0.34	-	-	-	-	mmu-mir-340	
MP-27	chr11	77681078-77681174,+	0.02	-	h|r	r	-	mmu-mir-144	
MP-28	chr11	77685721-77685807,+	1.19	h|m	h|r	h|r	h|r	mmu-mir-144	X65
MP-29	chr11	96515423-96515539,+	3	-	h|r	h	-	mmu-mir-152	
MP-30	chr11	96523412-96523475,+	0.04	-	-	-	-	mmu-mir-152	
MP-31	chr12	103597091-103597166,+	0.45	-	r	r	-	mmu-mir-345	
MP-32	chr12	104329614-104329696,+	1.47	-	h|r	h|r	r	mmu-mir-337	B202, X52
MP-33	chr12	104334262-104334328,+	0.18	m	r	r	r	mmu-mir-337	
MP-34	chr12	104342700-104342775,+	0.61	m	r	r	-	mmu-mir-136	
MP-35	chr12	104463492-104463576,+	1.89	m	h|r	h|r	h|r	mmu-mir-329	X61
MP-36	chr12	104463885-104463947,+	0.37	-	h|r	h|r	-	mmu-mir-329	
MP-37	chr12	104465432-104465507,+	0.41	m	h|r	h|r	r	mmu-mir-329	B210, X202
MP-38	chr12	104470892-104470977,+	0.91	m	h|r	h|r	h|r	mmu-mir-376b	
MP-39	chr12	104475507-104475588,+	0.89	m	h|r	h|r	-	mmu-mir-381	B212, X199
MP-40	chr12	104476303-104476376,+	1.03	h	h|r	h|r	-	mmu-mir-381	B213, X177
MP-41	chr12	104477505-104477568,+	0.19	-	h|r	h|r	-	mmu-mir-381	B214
MP-42	chr12	104483072-104483152,+	1.68	m	h|r	h|r	h|r	mmu-mir-134	B217, X23
MP-43	chr12	104487293-104487371,+	1.41	-	h|r	h|r	r	mmu-mir-154	B219, X161
MP-44	chr12	104490583-104490672,+	0.49	m	r	r	r	mmu-mir-409	
MP-45	chr12	104491592-104491670,+	0.76	h|m	h|r	h|r	h	mmu-mir-412	B222
MP-46	chr13	109978142-109978233,+	0.8	-	h|r	h|r	-	mmu-mir-449	
MP-47	chr14	56583977-56584048,+	0.02	-	r	r	r	mmu-mir-124a-1	
MP-48	chr15	82530668-82530751,+	0.87	-	r	-	-	mmu-mir-33	
MP-49	chr16	24680520-24680593,+	0.53	-	-	-	-	mmu-mir-28	
MP-50	chr16	77953522-77953626,+	0.74	-	h|r	h|r	h	mmu-mir-99a	
MP-51	chr17	16473418-16473539,+	0.58	-	r	r	-	mmu-mir-99b	
MP-52	chr18	62164083-62164186,-	0.17	-	r	r	-	mmu-mir-145	
MP-53	chr19	6053317-6053411,+	1.8	-	-	-	-	mmu-mir-192	
MP-54	chrX	5530237-5530320,-	0.86	-	h|r	h|r	-	mmu-mir-188	B122
MP-55	chrX	5544605-5544684,-	0.5	-	h|r	r	-	mmu-mir-188	
MP-56	chrX	45088040-45088119,-	0.24	h|m	h|r	h|r	-	mmu-mir-19b-2	X216
MP-57	chrX	45572242-45572319,-	1.19	-	h|r	h	h	mmu-mir-450	
MP-58	chrX	45572542-45572610,-	0.44	h	h|r	h|r	-	mmu-mir-450	X138
MP-59	chrX	45573646-45573730,-	0.42	h|m|r	h|r	h|r	h|r	mmu-mir-450	
MP-60	chrX	45578227-45578297,-	0.6	m|r	h|r	h|r	-	mmu-mir-322	X141
MP-61	chrX	59554721-59554798,-	0.92	m	r	r	-	mmu-mir-201	
MP-62	chrX	59563306-59563373,-	0.11	-	r	-	-	mmu-mir-201	
MP-63	chrX	63820617-63820683,-	0.04	h	h	h	-	mmu-mir-224	X154
MP-64	chrX	96740883-96740952,-	0.19	-	-	-	-	mmu-mir-384	
MP-65	chrX	104392971-104393058,-	1.05	-	r	-	-	mmu-mir-361	
MP-66	chrUn random	95645494-95645572,-	1.14	m	r	r	r	mmu-mir-292	
