# Supplementary material for: Identification of clustered microRNAs using an ab initio prediction method
Source: BMC Bioinformatics. 2005 Nov 7;6:267. doi: 10.1186/1471-2105-6-267 (PMC1315341; doi:10.1186/1471-2105-6-267)
Supplement: Additional File 3 — All predictions, rat. The same as the Additional file 1, but for the rat predictions. The genome assembly used for the coordinates is rn3. [file 1471-2105-6-267-S3.pdf]

Name	Chromosome	Genomic coordinates	SVM score	Cloning	Conservation			Closest miRNA	Other predictions
RP-1	chr1	56485513-56485591,+	0.03	-	h|m	m	-	rno-mir-99b	
RP-2	chr1	64351073-64351153,-	1.25	-	-	-	-	rno-mir-290	L290
RP-3	chr1	64351438-64351513,-	0.36	-	m	-	-	rno-mir-290	
RP-4	chr1	78621631-78621710,+	0.17	-	m	-	-	rno-mir-330	
RP-5	chr1	78623834-78623909,+	0.06	-	m	-	-	rno-mir-330	
RP-6	chr1	95669980-95670053,+	0.23	-	m	-	-	rno-mir-150	
RP-7	chr1	95670581-95670654,+	0.09	-	m	-	-	rno-mir-150	
RP-8	chr1	95677900-95677977,+	0.24	-	-	-	-	rno-mir-150	
RP-9	chr1	116101847-116101913,-	0.11	-	m	-	-	rno-mir-344	
RP-10	chr1	118729804-118729885,+	0.07	-	m	m	m	rno-mir-211	
RP-11	chr1	232943460-232943537,+	0.53	-	m	-	-	rno-mir-101b	
RP-12	chr1	238976094-238976179,-	1.08	-	h|m	m	-	rno-mir-107	
RP-13	chr2	44351863-44351934,+	0.09	-	m	-	-	rno-mir-449	
RP-14	chr3	4776371-4776446,+	0.02	-	m	m	-	rno-mir-126	
RP-15	chr3	8825965-8826040,-	0.12	-	m	m	-	rno-mir-219-2	
RP-16	chr3	8826355-8826426,-	0.12	-	h|m	-	-	rno-mir-219-2	
RP-17	chr3	8836204-8836279,-	0.13	-	h|m	m	m	rno-mir-219-2	
RP-18	chr3	18555202-18555275,+	0.31	-	m	m	-	rno-mir-181a	
RP-19	chr3	67845233-67845310,-	0.35	-	m	m	-	rno-mir-130a	
RP-20	chr3	67854618-67854691,-	0.06	-	m	-	-	rno-mir-130a	
RP-21	chr3	118900434-118900505,+	0.16	-	m	-	-	rno-mir-103-2	
RP-22	chr3	169917182-169917255,+	0.36	-	-	-	-	rno-mir-124a-3	
RP-23	chr5	74811172-74811258,-	0.42	-	m	-	-	rno-mir-32	
RP-24	chr5	122003543-122003652,-	4.13	-	-	-	-	rno-mir-101	
RP-25	chr5	141361542-141361623,-	0.64	-	m	-	-	rno-mir-30c-1	
RP-26	chr5	141373038-141373110,-	0.21	-	h|m	h|m	m	rno-mir-30e	
RP-27	chr5	172919255-172919345,-	0.1	-	h|m	h|m	h	rno-mir-200b	
RP-28	chr6	133180935-133181008,+	0.04	-	h|m	h|m	h|m	rno-mir-345	
RP-29	chr6	134176527-134176610,+	1.69	h	h|m	h|m	m	rno-mir-337	X52
RP-30	chr6	134183220-134183303,+	0.58	m	h|m	h|m	h|m	rno-mir-127	
RP-31	chr6	134216381-134216459,+	0.77	h|m	h|m	h|m	h|m	rno-mir-341	
RP-32	chr6	134394385-134394469,+	0.27	h|m	h|m	h|m	h|m	rno-mir-329	
RP-33	chr6	134401055-134401137,+	1.57	h	m	m	m	rno-mir-300	X61
RP-34	chr6	134401405-134401476,+	0.56	-	h|m	h|m	h	rno-mir-300	
RP-35	chr6	134403345-134403424,+	1.12	h	h|m	h|m	h|m	rno-mir-300	X202
RP-36	chr6	134409396-134409480,+	0.41	-	h|m	h|m	h|m	rno-mir-300	
RP-37	chr6	134409711-134409794,+	1.2	m	h|m	h|m	h|m	rno-mir-300	
RP-38	chr6	134410367-134410452,+	1.88	m	h|m	h|m	-	rno-mir-300	X205
RP-39	chr6	134410877-134410958,+	0.92	h|m	h|m	h|m	h	rno-mir-134	
RP-40	chr6	134413887-134413947,+	0.08	h	h|m	h|m	m	rno-mir-134	
RP-41	chr6	134414396-134414477,+	0.89	h	h|m	h|m	h|m	rno-mir-134	X199
RP-42	chr6	134415104-134415179,+	1.12	h	h|m	h|m	m	rno-mir-134	X177
RP-43	chr6	134416447-134416510,+	0.32	-	m	m	m	rno-mir-134	
RP-44	chr6	134416556-134416621,+	0.14	-	h|m	h|m	-	rno-mir-134	
RP-45	chr6	134421450-134421529,+	0.81	h|m	m	m	m	rno-mir-154	
RP-46	chr6	134422571-134422649,+	1.61	m	m	-	-	rno-mir-154	X23
RP-47	chr6	134426555-134426633,+	1.46	-	h|m	h|m	-	rno-mir-154	X161
RP-48	chr6	134427956-134428039,+	1.46	h|m	h|m	h|m	h|m	rno-mir-154	
RP-49	chr6	134429927-134430016,+	1.39	m	m	m	-	rno-mir-154	
RP-50	chr6	134430691-134430767,+	0.77	h|m	h|m	h|m	-	rno-mir-154	
RP-51	chr6	134430965-134431043,+	0.67	h|m	m	-	-	rno-mir-154	
RP-52	chr6	144523740-144523813,+	0.33	-	m	m	m	rno-mir-153	
RP-53	chr7	31103016-31103110,-	0.83	-	h|m	h|m	m	rno-mir-331	
RP-54	chr7	31113223-31113298,-	0.06	-	h|m	m	-	rno-mir-331	
RP-55	chr7	105855778-105855851,-	0.04	-	-	-	-	rno-mir-30d	
RP-56	chr7	105858098-105858171,-	0.03	-	-	-	-	rno-mir-30d	
RP-57	chr7	120613557-120613657,+	0.79	-	m	-	-	rno-mir-33	
RP-58	chr8	71573210-71573289,-	0.54	-	m	-	-	rno-mir-190	
RP-59	chr8	94715612-94715679,-	0.03	-	m	-	-	rno-mir-184	
RP-60	chr8	124000726-124000803,+	0.63	-	m	-	-	rno-mir-26a	
RP-61	chr8	124008840-124008920,+	0.59	-	m	-	-	rno-mir-26a	
RP-62	chr9	19403192-19403268,+	1	m|r	m	m	-	rno-mir-206	L133
RP-63	chr9	73886715-73886788,+	0.09	-	m	m	-	rno-mir-26b	
RP-64	chr10	20489742-20489826,+	0.34	-	m	m	-	rno-mir-218-2	
RP-65	chr10	62803973-62804046,+	0.24	-	m	-	-	rno-mir-22	

RP-66	chr10	64143143-64143210,+	0.56	h|m	m	-	-	rno-mir-144	B919, X65
RP-67	chr10	64148879-64148959,+	0.69	-	m	-	-	rno-mir-144	
RP-68	chr10	74887785-74887858,-	0.23	-	m	m	-	rno-mir-21	
RP-69	chr10	85614388-85614461,+	0.13	-	m	-	-	rno-mir-152	
RP-70	chr10	85620609-85620682,+	0.13	-	m	-	-	rno-mir-152	
RP-71	chr13	40923138-40923237,+	0.11	-	m	-	-	rno-mir-128a	
RP-72	chr13	40923354-40923429,+	0.06	-	h|m	-	-	rno-mir-128a	
RP-73	chr14	67635179-67635254,-	0.49	-	m	-	-	rno-mir-218-1	
RP-74	chr14	67646626-67646689,-	0.44	-	m	-	-	rno-mir-218-1	
RP-75	chr15	33061966-33062039,-	0.09	-	-	-	-	rno-mir-208	
RP-76	chr15	50851770-50851843,+	0.15	-	h|m	h|m	h|m	rno-mir-320	
RP-77	chr15	50859666-50859770,+	0.35	-	h|m	h|m	h	rno-mir-320	
RP-78	chr18	2182016-2182089,-	0.03	-	h|m	h|m	m	rno-mir-133a	
RP-79	chr18	2191671-2191757,-	1.89	m	h|m	h|m	-	rno-mir-133a	L203
RP-80	chr18	16238116-16238208,-	0.65	-	h|m	h|m	h|m	rno-mir-187	
RP-81	chr18	16240916-16241012,-	3.2	-	m	-	-	rno-mir-187	
RP-82	chr18	27074044-27074117,-	0	-	-	-	-	rno-mir-333	
RP-83	chr18	27076891-27076964,-	0.32	-	m	-	-	rno-mir-333	
RP-84	chr18	57689589-57689662,-	0.23	-	m	m	-	rno-mir-143	
RP-85	chr18	57690454-57690526,-	0.27	-	m	-	-	rno-mir-143	
RP-86	chr18	61576948-61577025,+	0.01	-	m	-	-	rno-mir-122a	
RP-87	chr18	61578429-61578490,+	0.63	-	m	-	-	rno-mir-122a	
RP-88	chr18	61584305-61584374,+	0.45	-	m	-	-	rno-mir-122a	
RP-89	chr19	25649163-25649264,-	0.57	-	h|m	h|m	h|m	rno-mir-23a	
RP-90	chr19	37421397-37421475,+	0.06	-	m	m	-	rno-mir-140	
RP-91	chr20	4972613-4972686,+	0.04	-	m	m	-	rno-mir-219-1	
RP-92	chrUn	25516733-25516880,-	1.55	-	m	-	-	-	
RP-93	chrX	31110201-31110285,-	0.1	-	m	-	-	rno-mir-448	
RP-94	chrX	41434223-41434298,+	0.03	-	m	m	m	rno-mir-98	
RP-95	chrX	91629279-91629352,-	0.39	-	h|m	h|m	m	rno-mir-421	
RP-96	chrX	91635942-91636015,-	0.03	-	m	-	-	rno-mir-421	
RP-97	chrX	91637005-91637072,-	1.05	h|m|r	m	m	-	rno-mir-421	X257
RP-98	chrX	93560933-93561017,-	0.29	-	m	m	-	rno-mir-325	
RP-99	chrX	139814084-139814170,-	0.88	h|m|r	m	m	-	rno-mir-19b-2	X211
RP-100	chrX	139814504-139814575,-	1.01	h|m|r	m	m	-	rno-mir-19b-2	X216
RP-101	chrX	140069623-140069701,-	0.53	h|m|r	h|m	h|m	-	rno-mir-322	B972
RP-102	chrX	140070350-140070436,-	2.3	-	m	-	-	rno-mir-322	
RP-103	chrX	140073303-140073373,-	0.78	m|r	m	m	-	rno-mir-322	B973, X141
RP-104	chrX	140079627-140079696,-	0.25	-	m	-	-	rno-mir-322	
RP-105	chrX	140080694-140080788,-	0.29	-	-	-	-	rno-mir-322	
