# Supplementary material for: Identification of clustered microRNAs using an ab initio prediction method
Source: BMC Bioinformatics. 2005 Nov 7;6:267. doi: 10.1186/1471-2105-6-267 (PMC1315341; doi:10.1186/1471-2105-6-267)
Supplement: Additional File 4 — All confirmed, human. Details of the confirmed miRNAs for human. The cloning frequency indicates the number of distinct small RNA cloned sequences found in the comprehensive cloning set that match our predicted pre-miRNA, "h", "m", and "r" corresponding to human, mouse, and rat, respectively. The secondary structure uses the text display from Mfold [32]. The last column gives the best homologs (within at most 5 mismatches) found in our confirmed predictions, in the false negative sets (Additional files 7, 8, and 9) and in Rfam7.1 (latest release at the time of publication). Notice MP-61 with good cloning evidence but an unusual position in the secondary structure. [file 1471-2105-6-267-S4.pdf]

Name	Arm	Mature miRNA sequence	Cloning frequency	Pre-miRNA genomic coordinates	Pre-miRNA secondary structure	SVM score	Homologs (mismatches<6)
HP-30	3	UGAAGGUCUACUGUGUGCCAG	2(h)	chr14, 100405160-100405230, +	U CAUUC U<br>GGGCUU GUACAUGGUAGGCUUUCAUU GU U<br>CCCGGA CGUGUGUCAUCUGGAAGUGG CA G<br>C CUUA- C	0.12	hsa-mir-493*(0)<br>RP-29(0)<br>MP-32(0)
HP-31	5	UCUUGGAGUAGGUCAUUGGGU	3(h)	chr14, 100420573-100420666, +	U - C UCU UA G A UCUAUUU<br>GA CU CUCCAGG UGGAG GGUCAUU GGUGG UCC \\<br>CU GA GAGGUUC ACCUC UCGGUAG UCACC GGG C<br>A A U UGU C- G - UGCAUUC	1.27	hsa-mir-432(0)
HP-33	3	AACACACCUGGUUAACCUCUU	2(h)	chr14, 100562875-100562954, +	CU UU UG UUC U AA<br>GGUAC GAAGAGAGGUU C GGU UGUUUC UU U<br>CUAUG CUUUUCUCCAA G CCA ACAAAG AG G<br>AC UU GU C-- C GA	1.29	HP-34(0)<br>hsa-mir-329-1(0)<br>mmu-mir-329(4)<br>rno-mir-329(4)
HP-34	3	AACACACCUGGUUAACCUCUU	2(h)	chr14, 100563199-100563264, +	UU UG UUC U AU<br>GAAGAGAGGUU C GGU UGUUUC UU U<br>CUUUUCUCCAA G CCA ACAAAG AG G<br>UU GU C-- C GA	0.25	HP-33(0)<br>hsa-mir-329-2(0)<br>mmu-mir-329(4)<br>rno-mir-329(4)
HP-35	3	UGAAACAUACACGGGAAACCU	1(h)	chr14, 100565724-100565804, +	C GU - C - UUUA<br>GAUACU GAAGGAGAGGUU CCGUGU UGU UUC UC \\<br>CUAUGA UUUUUUCUCCAA GGCACA ACA AAG AG U<br>- AG U - U UAUU	1.4	hsa-mir-494(0)<br>MP-35(0)<br>RP-33(0)
HP-37	3	AUCAUAGAGGAAAAUCCACG	1(h)	chr14, 100576159-100576238, +	U G A U -- GU<br>GGUAUU AAAA GU GAUUUUCCU CUAUGGUUA CGU \\<br>CUAUGA UUUU CA CUAAAAGGA GAUACUAAU GUA U<br>C G C - UG GU	1.31	hsa-mir-376a(0)<br>mmu-mir-376a(1)<br>RP-39(1)
HP-40	3	AAUCGUACAGGGUCAUCCACUU	3(h)	chr14, 100582545-100582628, +	U UG UUAU CCU UUUUG<br>UGGUACU GAGAGUGG CCCUGU GUUCG C<br>ACUAUGA UUUUCACC GGGACA UAAGC U<br>A CU UACU UGC UGUAC	1.28	MP-39(0)<br>RP-41(0)
HP-41	5	GGAGAAAUUAUCCUUGGUGUGU	2(h)	chr14, 100583411-100583488, +	UG A G - C UAU<br>AUACU AGGAGAAAUU UCCUUG UGUG UUCG UU \\<br>UAUGA UUUUCUUUAA AGGAAC AUAC AAGU AG U<br>GU C - U - UAU	1.17	MP-40(0)<br>RP-42(0)
HP-42	3	AUUCUGCAUUUUUAGCAAGU	1(h)	chr14, 100584748-100584838, +	AUUUU CUA U - U GA- C<br>CAUCAC GGGA CUUGUUAAAAA GCAGA UCU UU A<br>GUAGUG CUCU GAACGAUUUUU CGUCU AGA AG G<br>UAAUC AA- U A U ACC G	0.3	MP-41(1)<br>RP-43(1)
HP-44	3	AAUCAUACAGGGACAUCCAGU	1(h)	chr14, 100588536-100588615, +	U G UUA C - C AU<br>GGUACU GAAGA UGG UCCCUG UGUG UUCG UUA \\<br>CUAUGA UUUUU ACC AGGGAC AUAC AAGC AGU U<br>C G UAC - U - AU	1.11	hsa-mir-487(0)<br>HP-40(3)<br>MP-39(3)<br>RP-38/41(3)
HP-45	5	AGAGGCUGGCCGUGAUGAAUUC	1(m)	chr14, 100591505-100591585, +	U GU UG CU - A AUUCA<br>G ACU GAGAGAGG GGCCGUG AUGA UUCG \\<br>C UGA UUCUCUCC UCGGCAC UACU GAGC U<br>A UG UU UC A - GAAAC	1.41	hsa-mir-485(0)<br>mmu-mir-485(0)<br>MP-42(0)<br>RP-46(0)

HP-47	5	AGGUUACCCGAGCAACUUUG	3(h)	chr14, 100601390-100601468, +	U U GG A AC - CA UG<br>GGUAC CG GAG GGUU CCGAGCAAC UUUG UC \\<br>CUAUG GC UUC CCAA GGCUCGUUG AAGC AG G<br>A - UU C GU U -- CA	0.74	hsa-mir-409(0)<br>mmu-mir-409(0)<br>RP-50(0)
	3	CGAAUGUUGCUCGGUGAACCCC	1(h), 1(m)				
HP-48	3	UUCACCUGGUCCACUAGCC	1(m)	chr14, 100601548-100601614, +	A C U AA - U<br>GGAUGG UGGU GACCAG UGG AGUA AUUG U<br>CCUGCC AUCA CUGGUC ACU UCAU UAAU U<br>G C C -- G C	0.18	hsa-mir-412(0)<br>mmu-mir-412(0)
HP-49	3	AAUAUAACACAGAUGGCCUG	3(h), 1(m)	chr14, 100602002-100602081, +	C G UG A A C UUA<br>GGUAC UGAGAA AGGU UCUGUG UG GUUCG UU U<br>CCAUG ACUUUU UCCG AGACAC AU UAAGC AG U<br>- G GU A A - UAA	0.78	hsa-mir-410(0)<br>mmu-mir-410(0)<br>RN-8(0)
HP-56	3	AACUGGCCCUCAAAGUCCCGC	2(h)	chr16, 14305325-14305407, +	GU U GU G A AG UUAU<br>GUG CUCAGAA CGGG UUUGAGGGC AG UG U \\<br>UAC GGGUUUU GCCC AAACUCCCG UC AC A G<br>UG C UG G A CU UUUU	1.25	hsa-mir-193b(0)<br>mmu-mir-193(4)<br>rno-mir-193(4)
HP-61	5	AAACCGUUACCAUUACUGAGU	6(h), 5(m)	chr17, 24212514-24212583, -	U A GA A<br>UGGG AUGGCAAG AACCGUUACCAUUACUG G<br>ACCC UAUCGUUC UUGGUAAUGGUAAUGAU U<br>G A UC U	0.83	hsa/mmu/rno-mir-451(0)<br>MP-28(0)<br>RP-66(0)
HP-77	5	AAUCCUUGGAACCUAGGUGUGA	1(h), 1(m)	chrX, 49476605-49476676, +	- ACC A- UG AU<br>CCUCUU GAAUCCUUGGA UAGGUGUG G CU \\<br>GGAGAA CUUAGGAACUU AUCCACAC C GA U<br>A --- AA GU CU	0.32	hsa-mir-362(0)<br>MN-9(0)
HP-80	3	AUCAGCAAACAUUUAUUGUGUG	2(h)	chrX, 73289960-73290065, -	CCCAG ---- A -- CU- A A AUAAAU<br>CCUG GC CAU UAGUAGGC CAGUAAAUGUUU UU GAUGA \\<br>GGAC CG GUG AUCGUCCG GUUAUUUACAAA GA CUACU G<br>----- ACCU A AA UGU C - CAGUAA	2.01	HN-9(5)
HP-85	5	CGGGUGGAUCACGAUGCAAUUU	1(h)	chrX, 133028921-133029009, -	U GU CA A GAUGAGUA<br>GUUUUGC GUU CGGGUGGAU CG UGCAAUUUU U<br>CAGGACG CAA GUCUACCUA GC ACGUUAAAA C<br>C AU UG - AGAGGAUA	1.14	hsa-mir-363(0)<br>mmu-mir-363(0)<br>MN-8(0)<br>RP-99(0)
	3	AAUUGCACGGUAUCCAUCUGU	5(h), 1(m)				
HP-86	5	UUUUGCGAUGUGUUCCUAAU	1(h)	chrX, 133400068-133400148, -	U UUU U AAU<br>GAUGC AAACUAU UGCGA GUGUUCCUAAUAUGU \\<br>CUAUG UUUGAUA ACGUU UACAGGGGUUAUGUA A<br>U CUU U AAU	1.59	hsa-mir-450-1/2(0)<br>mmu-mir-450(0)<br>MP-58(0)<br>rno-mir-450(1)
HP-87	5	UAGCAGCGGGAACAGUUCUG	3(h)	chrX, 133405878-133405948, -	A U G GU AU<br>UGCCCU GCAGCGGGAACAGU CU CA GAGCG \\<br>AUGGGA CGUCGCCUUUGUUA GG GU CUCGU C<br>C U G -- GG	0.8	hsa-mir-503(0)<br>MP-60(1)<br>RP-103(1)
HP-88	5	AACUGUUUGCAGAGGAAACUGA	1(h)	chrX, 150798672-150798748, -	AA GU G A UUUGU<br>AGCACUUAC CU UUGCAGA GA ACUGAGAC \\<br>UCGUGAAUG GA AACGUCU CU UGACUCUG A<br>AA -- A C UAUCA	1.02	hsa-mir-452(0)<br>mmu-mir-452(0)<br>MP-63(0)
