# Supplementary material for: Identification of clustered microRNAs using an ab initio prediction method
Source: BMC Bioinformatics. 2005 Nov 7;6:267. doi: 10.1186/1471-2105-6-267 (PMC1315341; doi:10.1186/1471-2105-6-267)
Supplement: Additional File 5 — All confirmed, mouse. Same as the Additional file 4, but for mouse. Notice that the case MP-28 is identical to MP-61. [file 1471-2105-6-267-S5.pdf]

Name	Arm	Mature miRNA sequence	Cloning frequency	Pre-miRNA genomic coordinates	Pre-miRNA secondary structure	SVM score	Homologs (mismatches<6)
MP-5	3	AAUUGCACUUUAGCAAUGGUG	5(h), 3(m)	chr3, 128025558-128025632, +	`   -                CA     C    UUGAA`<br>`AGG CCGUCACUGUUGCUAA  UGCAA UCUG     U`<br>`UCC GGUAGUGGUAACGAUU  ACGUU AGGU     A`<br>`   A                UC     A    UAAAG`	0.78	hsa-mir-367(0)
MP-24	5	AUGGUGGCACGGAGUC	1(m)	chr10, 127120840-127120960, +	`           .-AAAA     -  -   U   U   ---       C    U    UA`<br>`CCUCUCUUCCC      CUAAA CA GGA CUA CAU   GGUGGCA GGAG CAGC  \`<br>`GGAGAGGAGGG      GAUUU GU CCU GAU GUG   CUAUCGU UCUC GUUG  G`<br>`           \ ----     U  A   U   C   UCC       -    U    UA`	0.9	hsa-mir-205(5)<br>mmu-miR-205(5)<br>rno-mir-205(5)<br>RN-3(5)
MP-28	5	AAACCGUUACCAUUACUGA	6(h), 5(m)	chr11, 77685721-77685807, +	`U     U-----     AU      GA                A`<br>` GGCAC      UGGGA  GGCGAG  AACCGUUACCAUUACUG G`<br>` CCGUG      ACCCU  UCGUUC  UUGGCAAUGGUAAUGAU U`<br>`A     UCAAAC     CG      UC                U`	1.19	hsa/mmu/rno-mir-451(0)<br>HP-61(0)<br>RP-66(0)
MP-33	3	AGGUCAGAGGUCGAUCCUGG	2(m)	chr12, 104334262-104334328, +	`      A      AC         U    G-   A`<br>`UGGGCC AGGGUC  CCUCUGAC CUGU  GCC \`<br>`AUCCGG UCCUAG  GGAGACUG GACA  UGG A`<br>`      G      CU         -    GA   G`	0.18	RP-30(0)
MP-34	5	GCUCGACUCAUGGUUUGAACC	3(m)	chr12, 104342700-104342775, +	`        A-  C    CUC      UG      UACU`<br>`UUGAACCA  AG UCGA   AUGGUU  AACCAU    U`<br>`AGCUUGGU  UC AGCU   UACCAA  UUGGUG    A`<br>`        CC  -    CAC      GU      CUUA`	0.61	mmu-mir-434(0)<br>RN-4(0)
	3	UUUGAACCAUCACUCGACUC	1(m)				
MP-35	3	UGAAACAUACACGGGAAACCU	1(m)	chr12, 104463492-104463576, +	`U       U            GU      -   C   -  UUUA`<br>` UGAUACU GAAGGAGAGGUU  CCGUGU UGU UUC UC    \`<br>` ACUAUGA UUUUUUCUCCAA  GGCACA ACA AAG AG    U`<br>`A       -            AG      U   -   U  UAUU`	1.89	hsa-mir-494(0)<br>HP-35(0)<br>RP-33(0)
MP-37	3	AAACAUUCGCGGUGCACUUCU	1(m)	chr12, 104465432-104465507, +	`U   U  U       U  -     --      -    UUU`<br>` GCU AA GAGAAGU GC CCGCG  UGUUUU UCGC   \`<br>` CGA UU UUCUUCA CG GGCGC  ACAAAG AGUG   A`<br>`A   C  U       -  U     UU      C    UAU`	0.41	HN-6(0)<br>RP-35(0)
MP-38	3	AACAUAGAGGAAAUUUCACG	2(m)	chr12, 104470892-104470977, +	`U   GU   U    G     UA     U         --   UU`<br>` UUG  AUU AAAA GUGGA  UUCCU CUAUGUUUA  UGC  \`<br>` AAC  UGA UUUU CACUU  AAGGA GAUACAAAU  GUG  U`<br>`A   UG   C    G     UA     -         UA   UU`	0.91	hsa-mir-368(2)<br>RP-37(0)
MP-39	3	AAUCGUACAGGGUCAUCCACU	3(m)	chr12, 104475507-104475588, +	`U      UG        UUAU      CCUC    - UUCA`<br>` GGUACU  GAGAGUGG    CCCUGU    UUCG C    \`<br>` CUAUGA  UUUUCACC    GGGACA    AAGC G    C`<br>`A      CU        UACU      UGCU    C UACU`	0.89	HP-40(0)<br>RP-41(0)
MP-40	5	GGAGAAAUUAUCCUUGGUGUGU	2(h)	chr12, 104476303-104476376, +	`    UG                 G    UUGGC  UU`<br>`UACU  AGGAGAAAUUAUCCUUG UGUG     UC  \`<br>`AUGA  UUUUCUUUAAUAGGAAC AUAC     AG  U`<br>`    GU                 -    UAAGU  GU`	1.03	HP-41(0)<br>RP-42(0)
MP-42	5	AGAGGCUGGCCGUGAUGAAUUC	1(m)	chr12, 104483072-104483152, +	`U      U         CU       -    A    AUUCA`<br>` GAUACU GGAGAGAGG  GGCCGUG AUGA UUCG     \`<br>` CUGUGA CUUCUCUCC  UCGGCAC UACU GAGC     U`<br>`A      U         UC       A    -    AAAUC`	1.68	mmu-mir-485(0)<br>hsa-mir-485(0)<br>HP-45(0)<br>RP-46(0)

MP-44	5	AAGGGAUUCUGAUGUUGGUCACA	6(m)	chr12, 104490583-104490672, +	A U AAG A G ---------- C<br>GCCA AA CAGAG GGAUUCUG UGUU GUCAC ACUC \<br>CGGU UU GUCUC CCUAAGAC ACAA CGGUG UGAG A<br>A C AUA - G AGUAAAAUUU A	0.49	RP-49(0)
MP-45	5	AGAUCGACCGUGUUAUAUUCG	2(h), 1(m)	chr12, 104491592-104491670, +	U AG UA CUU C<br>GGUACU GA GGAGAUCGACCGUGU UAUUCG GG U<br>CUAUGA CU UUUCUAGUUGGUACA AUAAGC UC G<br>- CU UA U-- A	0.76	hsa-mir-369(0)<br>RP-51(0)
	3	AAUAAUACAUGGUUGAUCUU	2(h), 1(m)				
MP-56	5	CAAAGUGCUCAUAGUGCAGGUA	5(h), 6(m)	chrX, 45088040-45088119, -	C U CA- G G UUUUUA<br>CUAG AGUGC AAGUGCUCAUA UGCAG UAG \<br>GGUC UCAUG UUCACGAGUGU ACGUC AUC U<br>- C AUC G - UCACCA	0.24	hsa-mir-20b(0)<br>HN-14(0)<br>RP-100(0)
MP-58	5	UUUUGCGAUGUGUUCCUAAU	1(h)	chrX, 45572542-45572610, -	UUU U UACU<br>GAACUAU UGCGA GUGUUCCUAAUAUG \<br>CUUGGUA ACGUU CGUAGGGGUUAUAU A<br>CUU U GAGU	0.44	hsa-mir-450-1/2(0)<br>mmu-mir-450(0)<br>rno-mir-450(0)<br>HP-86(0)
MP-59	5	CUCGGGGAUCAUCAUGUCACG	2(h), 1(m), 2(r)	chrX, 45573646-45573730, -	A GUC GG UCA A- C<br>GG UCUCAGAC UCGG AUCA UGUCACGAG UAC A<br>CC AGGGUCUG AGUC UAGU ACAGUGUUC GUG C<br>G GAA AA UAG CC U	0.42	RP-101(0)
	3	UGUGACAGAUUGAUAACUGAAA	1(h), 1(m)				
MP-60	5	UAGCAGCGGGAACAGUACUGC	6(m),4(r)	chrX, 45578227-45578297, -	UA C G UGA GUU<br>UGCCC GCAG GGGAACAGUACU CAG GU \<br>AUGGG CGUC CCUUUGUUAUGA GUC CG U<br>UC A G C-- UGG	0.6	hsa-mir-503(1)<br>HP-87(1)<br>RP-103(0)
MP-61	3	CUUGGUACAUCUUUGAGUGA	3(m)	chrX, 59554721-59554798, -	A A UG CC UUAAC<br>GUGUG UGU UCACU AGGAUGUACCA CAU A<br>CACAC AUA AGUGA UUCUACAUGGU GUA G<br>A G GU UC CAAAG	0.92	
MP-63	5	AACUGUUUGCAGAGGAAACUGA	1(h)	chrX, 63820617-63820683, -	UUACAA G G A UUUAU<br>CU UUUGCAGA GA ACUGAGAC \<br>GA AAACGUCU CU UGACUCUG A<br>AAUG-- G A C UAUCA	0.04	mmu-mir-452(0)<br>hsa-mir-452(0)<br>HP-88(0)
MP-66	5	GAUCAAAGUGGAGGCCCUCUC	4(m)	chrUn_random, 95645494-95645572, -	UGU U G C C- GG<br>ACAUACAG CGA CAAAGUGGA GC CU UCCGC C<br>UGUGUGUC GUU GUUUUACCU CG GA GGGCG U<br>UCU U A U AA GU	1.14	mmu-mir-291(1)<br>rno-mir-291(1)
	3	AAAGUGCAUCCAUUUUGUUUGUC	2(m)				mmu-mir-294(3)
