# Supplementary material for: Identification of clustered microRNAs using an ab initio prediction method
Source: BMC Bioinformatics. 2005 Nov 7;6:267. doi: 10.1186/1471-2105-6-267 (PMC1315341; doi:10.1186/1471-2105-6-267)
Supplement: Additional File 6 — All confirmed, rat. Same as the Additional file 4, but for rat. RP-66 is identical to HP-61 and MP-28. [file 1471-2105-6-267-S6.pdf]

Name	Arm	Mature miRNA sequence	Cloning frequency	Pre-miRNA genomic coordinates	Pre-miRNA secondary structure	SVM score	Homologs (mismatches<6)
RP-29	3	UGAAGGUCUACUGUGUGCCAG	2(h)	chr6, 134176527-134176610, +	CUC U CAU U<br>GGC CAGGGCCU GUACAUGGUAGGCUUUCAUU UGU U<br>CCG GUCCCGGA CGUGUGUCAUCUGGAAGUGG ACA G<br>U-- C CUU C	1.69	hsa-mir-493*(0)<br>HP-30(0)<br>MP-32(0)
RP-30	3	AGGUCAGAGGUCGAUCCUGG	2(m)	chr6, 134183220-134183303, +	UG C A AC U G- A<br>CCC GGG UGGGCC AGGGUC CCUCUGAC CUGU GCC \\<br>GGG CUC AUCCGG UCCUAG GGAGACUG GACA UGG A<br>GU - G CU - GA G	0.58	MP-33(0)
RP-31	3	GCCUGCUGGGGUGGAACCUGG	3(h),3(m)	chr6, 134216381-134216459, +	G CA GU U U A GC<br>AGACAGA AGACCAGGU C CUC GCAG UAC CA \\<br>UCUGUCU UUUGGUCCA G GGG CGUC GUG GU U<br>G AG UG U C A AC	0.77	hsa-mir-370(0)<br>mmu-mir-370(0)
RP-32	5	UGGUAGACUAUGGAACGUA	3(h),4(m)	chr6, 134394385-134394469, +	U UUC C G A GA - UUAU<br>GG CUG AGAG UGGU GACUAUG ACGUAGG CG \\<br>CC GAC UCUC AUCA CUGGUAC UGUAUCC GU G<br>A UAU - A C AA A CUUU	0.27	hsa-mir-379(0)<br>mmu-mir-379(0)
RP-33	3	UGAAACAUACACGGGAAACCU	1(h)	chr6, 134401055-134401137, +	U U GU - C - UUUA<br>GAUACU GAAGGAGAGGUU CCGUGU UGU UUC UC \\<br>CUAUGA UUUUUUCUCCAA GGCACA ACA AAG AG U<br>A - AG U - U UAUU	1.57	hsa-mir-494(0)<br>HP-35(0)<br>MP-35(0)
RP-35	3	AAACAUUCGCGGUGCACUUCU	1(m)	chr6, 134403345-134403424, +	U U U - -- - UUU<br>GGUGCU AA GAGAAGU GC CCGCG UGUUUU UCGC \\<br>CUACGA UU UUCUUCA CG GGCGC ACAAAG AGUG A<br>C U - U UU C UAU	1.12	HN-6(0)<br>MP-37(0)
RP-37	3	AACAUAGAGGAAAUUUCACG	2(m)	chr6, 134409711-134409794, +	U GU U G UA U UGGUAU<br>UG AUU AAAA GUGGA UUCCU CUAUGUUUA \\<br>AC UGA UUUU CACUU AAGGA GAUACAAAU U<br>A UG C G UA - UAGUAU	1.2	hsa-mir-368(2)<br>MP-38(0)
RP-38	5	GUGGAUAUUCCUUCUAUGGUU	2(m)	chr6, 134410367-134410452, +	U U A U CGUGCU<br>UUGGUAUU AAAAGGUGGAU UUCCU CUAUGGUUA \\<br>AACUAUGA UUUUUCACCUA AAGGA GAUACUAAU U<br>A C C - AGGUCC	1.88	
	3	AUCAUAGAGGAACAUCCACU	1(m)				mmu-mir-376b(0)<br>hsa-mir-376a(2)
RP-39	5	GGUAGAUUCUCCUUCUAUGAG	1(h),1(m)	chr6, 134410877-134410958, +	U U G A C U GUACAAUAU<br>GAUAUU AAAA GU GAUU UCCU CUAUGA \\<br>CUAUGA UUUU CA CUAA AGGA GAUGCU U<br>A C G C A - AAUCAGUAA	0.92	hsa-mir-376a(1)<br>mmu-mir-376a(0)<br>HP-37(1)
	3	AUCGUAGAGGAAAAUCCACG	2(m)				
RP-40	3	UAUACAAGGGCAAGCUCUC	1(h)	chr6, 134413887-134413947, +	C G U G UUA<br>AG GAG UUGCCCUU GUAUAUUC GU U<br>UC CUC AACGGGAA CAUAUAGG UA U<br>U G - G CAG	0.08	hsa/mmu-mir-381(0)<br>mmu/rno-mir-300(1)
RP-41	3	AAUCGUACAGGGUCAUCCACU	3(h)	chr6, 134414396-134414477, +	U UG UUAU CCUC - UUCA<br>GGUACU GAGAGUGG CCCUGU UUCG C \\<br>CUAUGA UUUUCACC GGGACA AAGC G C<br>A CU UACU UGCU C UAUU	0.89	HP-40(0)<br>MP-39(0)
RP-42	5	GGAGAAAUUAUCCUUGGUGUGU	2(h)	chr6, 134415104-134415179, +	G - CU U<br>AUACUUGAGGAGAAAUUAUCCUUG UGUG UUUG C U<br>UAUGAGCUUUUCUUUAAUGGGAAC AUAC AAGU G U<br>- U AG U	1.12	HP-41(0)<br>MP-40(0)
RP-45	5	GAAGUUGUUCGUGGUGGAUUC	1(h), 2(m)	chr6, 134421450-134421529, +	U A- UG CU C<br>GGUACU GAAGAGA GUUGUUCGUGG GAUUCG UUA U<br>CCAUGA CUUUUUU CAACAGGCACU CUAAGC AGU U<br>- CA UA -- G	0.81	hsa-mir-382(0)<br>mmu-mir-382(0)

<table>
<tr><td rowspan="1">RP-46</td><td>5</td><td>AGAGGCUGGCCGUGAUGAAUUC</td><td>1(m)</td><td>chr6, 134422571-134422649, +</td><td>   U   CU   -   A   AUUCA
GAUACU GGAGAGAGG GGCCGUG AUGA UUCG   \
CUGUGA CUUCUCUCC UCGGCAC UACU GAGC   U
      U   UC   A   -   AAAUC</td><td>1.61</td><td>hsa-mir-485(0)
mmu-mir-485(0)
HP-45(0)
MP-42(0)</td></tr>
<tr><td rowspan="2">RP-48</td><td>5</td><td>AGAGGUUGCCCUUGGUGAAUU</td><td>1(h)</td><td rowspan="2">chr6, 134427956-134428039, +</td><td rowspan="2">U   U UG G   C   -   A   - UUUA
 UGAU CU A CAGAGGUUGCC UUG GUGA UUCG C   \
 ACUA GA U GUUUUCAACGG AAC CACU AAGU G   U
A   U GU G   A   A   -   U UAGU</td><td rowspan="2">1.46</td><td>HP-45(4),MP-42(4)
RP-46(4)</td></tr>
<tr><td>3</td><td>AAUCACACAAAGGCAACUUUU</td><td>3(h),1(m)</td><td>hsa-mir-377(1)
mmu-mir-377(1)</td></tr>
<tr><td>RP-49</td><td>5</td><td>AAGGGAUUCUGAUGUUGGUCACA</td><td>6(m)</td><td>chr6, 134429927-134430016, +</td><td>   A U   AAG   A   G   ACUCCAAGAA
GCCA AA CAGAG   GGAUUCUG UGUU GUCAC   \
CGGU UU GUCUC   CCUAAGAC ACAA CGGUG   U
    A C   AUA   -   G   AGUAAAAUUU</td><td>1.39</td><td>MP-44(0)</td></tr>
<tr><td rowspan="2">RP-50</td><td>5</td><td>AGGUUACCCGAGCAACUUUG</td><td>3(h)</td><td rowspan="2">chr6, 134430691-134430767, +</td><td rowspan="2">   U   A   AC   -   CA UG
GGUAC CGGAGAG GGUU CCGAGCAAC UUUG UC \
CUAUG GCUUUUC CCAA GGCUCGUUG AAGC AG G
     -       C   GU   U   -- GA</td><td rowspan="2">0.77</td><td rowspan="2">hsa-mir-409(0)
mmu-mir-409(0)
HP-47(0)</td></tr>
<tr><td>3</td><td>AAUGUUGCUCGGUGAACCCC</td><td>1(h),1(m)</td></tr>
<tr><td rowspan="2">RP-51</td><td>5</td><td>AGAUCGACCGUGUUAUAUUCG</td><td>2(h),1(m)</td><td rowspan="2">chr6, 134430965-134431043, +</td><td rowspan="2">   U AG   UA   CUU C
GGUACU GA GGAGAUCGACCGUGU UAUUCG   AG U
CUAUGA CU UUUCUAGUUGGUACA AUAAGC   UC G
      - CU   UA   U-- A</td><td rowspan="2">0.67</td><td rowspan="2">hsa-mir-369(0)
MP-45(0)</td></tr>
<tr><td>3</td><td>AAUAAUACAUGGUUGAUCUU</td><td>2(h),1(m)</td></tr>
<tr><td>RP-62</td><td>3</td><td>UUUGGUCCCCUUCAACCAGC</td><td>1(m),1(r)</td><td>chr9, 19403192-19403268, +</td><td>   C   CA C A   U G   C
CCCUGCU UGGCUGGU AA GG ACCAAG CC UCUU \
GGGACGA AUCGACCA UU CC UGGUUU GG AGAG C
       C   AC C C   - -   U</td><td>1</td><td>hsa/mmu/rno-
mir-133a(0)
hsa/mmu-mir-133b(0)</td></tr>
<tr><td>RP-66</td><td>5</td><td>AAACCGUUACCAUUACUGAG</td><td>6(h),5(m)</td><td>chr10, 64143143-64143210, +</td><td>U   AU   GA   A
 GGGA GGCGAG AACCGUUACCAUUACUG G
 CCCU UCGUUC UUGGUAAUGGUAAUGAU U
A   CG   UC   U</td><td>0.56</td><td>hsa/mmu/rno-
mir-451(0)
HP-61(0)
MP-28(0)</td></tr>
<tr><td>RP-79</td><td>5</td><td>GCACAUACUUCUUUAUGUACCC</td><td>1(m)</td><td>chr18, 2191671-2191757, -</td><td>   C   AC   UGAACA
UGCCUACUCAGAG ACAUACUUCUUUAUGU CCAUA   U
AUGGAUGGGUUUU UGUGUGAAGAAAUGUA GGUAU   A
             A   A-   CGUAAG</td><td>1.89</td><td>mmu-mir-1-2*(0)</td></tr>
<tr><td>RP-97</td><td>5</td><td>AUAUAAUACAACCUGCUAAGU</td><td>3(h),5(m),
2(r)</td><td>chrX, 91637005-91637072, -</td><td>   UG AU   C   U
CUCGGA G AUAAUACAAC UGCUAAGUGU C
GAGCCU U UAUUAUGUUG ACGAUUCACG U
      GU AU   C   A</td><td>1.05</td><td>hsa-mir-374(2)</td></tr>
<tr><td rowspan="2">RP-99</td><td>5</td><td>CGGGUGGAUCACGAUGCAAUUU</td><td>1(h)</td><td rowspan="2">chrX, 139814084-139814170, -</td><td rowspan="2">   U   AU   CA A   GA--   GA
UUUUGC GUU CGGGUGGAU CG UGCAAUUUU   UUA G
AGGACG CAA GUCUACCUA GC ACGUUAAAG   GGU U
      C   AU   UG -   AGAG   AA</td><td rowspan="2">0.88</td><td rowspan="2">hsa-mir-363(0)
mmu-mir-363(0)
HP-85(0)
MN-8(0)</td></tr>
<tr><td>3</td><td>AAUUGCACGGUAUCCAUCUGU</td><td>5(h),1(m),
1(r)</td></tr>
<tr><td rowspan="2">RP-100</td><td>5</td><td>CAAAGUGCUCAUAGUGCAGGU</td><td>4(h),5(m)</td><td rowspan="2">chrX, 139814504-139814575, -</td><td rowspan="2">GU   -   G   G   - UUU
  AGUGCCA AAGUGCUCAUA UGCAG UAG GU   G
  UCAUGGU UUCACGAGUGU ACGUC AUC CA   C
C-   C   G   -   U CGU</td><td rowspan="2">1.01</td><td rowspan="2">hsa-mir-20b(0)
HN-14(0)
MP-56(0)</td></tr>
<tr><td>3</td><td>ACUGCAGUGUGAGCACUUCUG</td><td>3(r)</td></tr>
<tr><td rowspan="2">RP-101</td><td>5</td><td>CUCGGGGAUCAUCAUGUCACG</td><td>2(h),1(m),
2(r)</td><td rowspan="2">chrX, 140069623-140069701, -</td><td rowspan="2">   GUC   GG   UCA   AUACCA
UCUCAGAC   UCGG AUCA   UGUCACGAG   \
AGGGUCUG   AGUC UAGU   ACAGUGUUC   C
        GAA   AA   UAG   ACGCGU</td><td rowspan="2">0.53</td><td rowspan="2">MP-59(0)</td></tr>
<tr><td>3</td><td>UGUGACAGAUUGAUAACUGAA</td><td>1(h),1(m)</td></tr>
<tr><td>RP-103</td><td>5</td><td>UAGCAGCGGGAACAGUACUG</td><td>6(m),4(r)</td><td>chrX, 140073303-140073373, -</td><td>   UA   G   UGA GUU
UGCCC GCAGCGGGAACAGUACU CAG   GU   \
AUGGG CGUCGCCUUUGUUAUGA GUC   CG   U
     UC   G   C-- UGG</td><td>0.78</td><td>hsa-mir-503(1)
HP-87(1)
MP-60(0)</td></tr>
</table>
