# Supplementary material for: Identification of clustered microRNAs using an ab initio prediction method
Source: BMC Bioinformatics. 2005 Nov 7;6:267. doi: 10.1186/1471-2105-6-267 (PMC1315341; doi:10.1186/1471-2105-6-267)
Supplement: Additional File 7 — False negatives, human. Set of false negatives for human, i.e. stem loop candidates with a negative SVM score but which have a cloning evidence. The layout is identical to the table from the Additional files 4, 5, and 6. The Additional column "Other predictions" is filled as in the additional files 1, 2, and 3. [file 1471-2105-6-267-S7.pdf]

Name	Arm	Mature miRNA sequence	Cloning frequency	Pre-miRNA genomic coordinates	Pre-miRNA secondary structure	SVM score	Homologs (mismatches<6)	Other predictions
HN-1	3	ACCAACAUGGUGAAACCCU	1(m)	chr3, 37995864-37995931, +	UG UGGA - A- AUUUCA<br>GAGG GG UCACC UG GGUCAGG \<br>CUCU CC AGUGG AC CCAGUCC A<br>GU CAA- U AA GACCAG	-1.26	HN-3(0)	
HN-2	5	CAGGGUUUCACCAUGUUGG	1(m)	chr3, 52284705-52284776, -	GUA A UUCAC UU CU CU<br>GAGGC GGGU CAUG GGUCAGG GGU \<br>CUCCG CCCA GUGC CUAGUCC UCA U<br>CGA - UCUA- U- -- AG	-1.9	hsa-mir-196a(4)<br>mmu-miR-196a(4)<br>rno-mir-196a(4)	
HN-3	3	ACCAACAUGGUGAAACCCU	1(m)	chr4, 8133205-8133274, -	U UGGA UGA-- A-- C<br>GAGGCAGG UCAUU GGUCAGG GUU \<br>CUCUGUCC AGUGG CCAGUCC CAG A<br>A CAA- UACAA GAC A	-2.01	HN-1(0)	
HN-4	3	ACCAGGAGGCUGAGGCCCC	2(h)	chr14, 100411130-100411185, +	U ACC- G CU<br>GAGGGGUCUC GCCUCU CA GA \<br>CUCCCCGGAG CGGAGG GU CU C<br>U ACCA A UU	-1.42	MN-6(1)<br>RN-2(1)	
HN-5	5	UAGUAGACCGUAUAGCGUAC	1(h), 2(m)	chr14, 100559430-100559483, +	A AUA C UAU<br>UAGU GACCGU GCGUACG UU \<br>AUCA CUGGCA UGUAUGC AG C<br>C CAA - UGU	-0.78	mmu-mir-411*(0)<br>RN-5(0)	
HN-6	3	AAACAUUCGCGGUGCACUUCU	1(m)	chr14, 100568085-100568144, +	U - U--- U UUU<br>GAGAAGU GC CCGUG UUUU UCGC \<br>UUCUUCA CG GGCGC AAAG AGUG A<br>- U UUAC C UUU	-1.63	MP-37(0)<br>RP-35(0)	B210, X202
HN-7	5	UGGUGGGCCGCAGAACAUGUG	1 (h)	chr14, 100576319-100576381, +	A GCC A - - A<br>AAAG UGGUGG GCAGA CAUGUG C UG G<br>UUUC ACUACC CGUCU GUAUAC G GC U<br>C AGU - C U U	-0.13	hsa-mir-380-5p(5)<br>mmu-miR-380-5p(5)	
	3	UAUGUCUGCUGACCAUCAC	1 (h)					
HN-8	5	AGGUUGUCCGUGGUGAGUUCGC	1 (h)	chr14, 100592317-100592383, +	U GUGAGUUC U<br>GGAGGGAGGUUG CCGUG GCAUUAU \<br>CUUUUCUCCAGC GGCAC CGUAGUA U<br>U AUAACC-- A	-1.17	hsa-mir-453-3P(0)<br>RP-45/48(5)	ANMH-52
HN-9	5	GCAGCACACUGUGGUUUG	2(m)	chr17, 6861984-6862041, -	G C U -- AC<br>AGCA CACA UGUGGUUUG AC GGC U<br>UUGU GUGU ACACCAAAC UG CCG G<br>G C C CA GU	-0.06	hsa-mir-497(0)<br>MN-5(0)<br>RN-10(0)	X43, ANMH64
HN-10	3	CAGUGCAAUGAUAUUGUCAAAGC	3(h)	chr22, 20331820-20331903, +	- G C G--- A GUGCU<br>GCUGG CC CAGGUGCU UGACGA GUUGCACU CU \<br>CGACC GG GUCUACGA ACUGUU UAACGUGA GA C<br>A - A AUAG C AGAGU	-0.75	hsa/mmu/rno-mir-301(2)	X220,<br>ANMH76
HN-11	5	UAAUCCUUGCUACCUGGGUGAG	1 (h)	chrX, 49476075-49476158, +	CCC U- UAC UG AGAG UGUC<br>GCUC CUCUC AAUCCUUGC C GGUG UGC \<br>CGAG GAGAG UUAGGAACG G CCAC ACG U<br>AGC UC --- GU GUA- UAAG	-0.33	hsa-mir-500(0)<br>HN-12-5(0)	
HN-12	5	UAAUCCUUGCUACCUGGGUGAG	1 (h)	chrX, 49478315-49478394, +	CUCU- UAC AGAG UUUC<br>UCCCCCU AAUCCUUGC CUGGGUG UGC \<br>AGGGGGA UUAGGAACG GACCCAC ACG U<br>ACGUC --- GUG- UAAG	-0.33	hsa-mir-500(0)<br>HN-11-5(0)	

HN-13	5	UACCCAUUGCAUAUCGGAGUUG	2(h)	chrX, 49480881-49480976, +	U ACU- C CAUAC- U C U GAAUU<br>GA GCUC UUCUCC CCAU GCAUAU GGAG UGU \<br>CU CGAG GGGAGG GGUA CGUGUG CCUC ACA C<br>A GUUC U ACAUUA - U C AAACU	-0.55		
HN-14	5	CAAAGUGCUCAUAGUGCAGGUA	5(h), 6(m)	chrX, 133029352-133029435, -	U U CA- G GU- UUG<br>GUCC AG AGUAC AAGUGCUCAUA UGCAG AGUU \<br>UAGG UC UCAUG UUCACGGGUAU AUGUC UCAG G<br>U - ACC G AUC UAC	-0.91	hsa-mir-20b(0)<br>MP-56(0)<br>RP-100(0)	X216
	3	ACUGUAGUAUGGGCACUUCCAG	1 (h)					
