# Supplementary material for: Identification of clustered microRNAs using an ab initio prediction method
Source: BMC Bioinformatics. 2005 Nov 7;6:267. doi: 10.1186/1471-2105-6-267 (PMC1315341; doi:10.1186/1471-2105-6-267)
Supplement: Additional File 8 — False negatives, mouse. Set of false negatives for mouse, similar to the Additional file 7. [file 1471-2105-6-267-S8.pdf]

<table>
<tr><th>Name</th><th>Arm</th><th>Mature miRNA sequence</th><th>Cloning frequency</th><th>Pre-miRNA genomic coordinates</th><th>Pre-miRNA secondary structure</th><th>SVM score</th><th>Homologs (mismatches<6)</th><th>Other predictions</th></tr>
<tr><td>MN-1</td><td>3</td><td>ACUUAGCUCACUUCUU</td><td>1(m)</td><td>chr1, 23518215-23518296, +</td><td>   AAAG   AG   GG   ---   ACA--   UCCU
AGUGAG   GGG AAAGG AGA   GAG   UGAA   \
UCACUU   CUC UUUCC UCU   CUC   ACUU   A
      GA--   GU   UU   UCA   GAUUC   CCAG</td><td>-1.75</td><td>hsa/mmu/rno-mir-27b(5)
hsa/rno-mir-151(5)
mmu/rno-mir-329(5)
HP-33/34(5)</td><td>B463</td></tr>
<tr><td>MN-2</td><td>3</td><td>UAAGUGCUUCCAUGUUUUAG</td><td>1(h), 1(m)</td><td>chr3, 128025063-128025120, +</td><td>   UU   AU   U G- UC
ACU AACAUGGGA GCUU CU UC \
UGA UUGUACCUU UGAA GA AG A
   UU   CG   U GA CU</td><td>-0.96</td><td>hsa-mir-302b(0)
hsa-mir-302a/c/d(1)
mmu-mir-302(1)</td><td></td></tr>
<tr><td>MN-3</td><td>5</td><td>ACAUUCAUUGUUGUCGGUGGGU</td><td>1(m)</td><td>chr8, 83449175-83449233, -</td><td>   G UG   GUGA AG
ACAUUCAUU U UCGGUGGGUU   GG G
UGUAAGUAG G GGCCACCCAG   CC C
         G --   A--- GA</td><td>-0.6</td><td>hsa-mir-181d(0)
hsa/mmu/rno-
mir-181b-1/2(1)</td><td>X74</td></tr>
<tr><td>MN-4</td><td>3</td><td>AGCCAUCUCUCCAGCC</td><td>1(h)</td><td>chr9, 106092117-106092176, +</td><td>   - AC A--   AGG C
GCUGGGGCUGG GA UG   GCUC   UC U
CGGCCCCGACC CU AC   CGAG   GG C
          U CU CGA   AA- G</td><td>-1.44</td><td>hsa-mir-197(4)
rno-mir-349(4)</td><td></td></tr>
<tr><td>MN-5</td><td>5</td><td>AGCAGCACACUGUGGUUUGUA</td><td>2(m)</td><td>chr11, 69847287-69847344, +</td><td>   G   C   U --   AC
AGCA CACA UGUGGUUUG AC GGC U
UUGU GUGU ACACCAAAC UG CCG G
    G   C   C CA   GU</td><td>-0.06</td><td>hsa-mir-497(0)
HN-9(0)
RN-10(0)</td><td>X43</td></tr>
<tr><td>MN-6</td><td>3</td><td>ACCAGGAGGCUGAGGUCCCUU</td><td>1(m)</td><td>chr12, 104334502-104334581, +</td><td>   U   -   U   AUCCA A   U
GGGUC CCU UGAGGGGCCUC GCCUCU   GG UUAUG U
UCCGG GGA AUUCCCUGGAG CGGAGG   CC AGUAU U
     C   C   U   A---- -   U</td><td>-0.01</td><td>HN-4(1)
RN-2(0)</td><td></td></tr>
<tr><td>MN-7</td><td>3</td><td>GGCUGCAGCGUGAUCGCCUG</td><td>2(m)</td><td>chr12, 104465268-104465345, +</td><td>   C   -   A-- -   GAG   - AG
CCUG GUG GAGCGGGC   CA GCUGU   AGCCCC CU G
GGAC CGC CUCGUCCG   GU CGACG   UCGGGG GA U
    A   A   CUA G   ---   C CA</td><td>-1.58</td><td>RN-7(0)</td><td></td></tr>
<tr><td>MN-8</td><td>3</td><td>AAUUGCACGGUAUCCAUCU</td><td>1(h)</td><td>chrX, 45087626-45087688, -</td><td>   - A A   GG GGUG
CAGGUGGA AC CG UGCAAUUUU UU   U
GUCUACCU UG GC ACGUUAAAA AG   A
        A - -   GG GAUA</td><td>-0.69</td><td>mmu-mir-363(0)
hsa-mir-363(0)
HP-85(0)
RP-99(0)</td><td>B343, X211</td></tr>
<tr><td>MN-9</td><td>5</td><td>AAUCCUUGGAACCUAGGUGU</td><td>1(m)</td><td>chrX, 5530957-5531028, -</td><td>   C-   ACC   AA   U U
CCUCU GAAUCCUUGGA   UAGGUGUG UGC GC U
GGAGA CUUAGGAACUU   GUCCACAC ACG UG C
     AA   ---   A-   - A</td><td>-0.61</td><td>hsa-mir-362(0)
HP-77(0)</td><td>B121, X144</td></tr>
<tr><td rowspan="2">MN-10</td><td>5</td><td>CAUGCCUUGAGUGUAGGACCG</td><td>1(h), 1(m)</td><td rowspan="2">chrX, 5537395-5537461, -</td><td rowspan="2">   C U   A   A   CC UGACAU
UCUU CA GCCUUG GUGU GGA GU   \
AGGA GU CGGAAC CACA CCU CA   C
    C U   C   C   CC UUAAUU</td><td>-0.13</td><td></td><td rowspan="2"></td></tr>
<tr><td>3</td><td>CCUCCCACACCCAAGGCUUGC</td><td>1(h),2(m),1(r)</td><td>-0.13</td><td></td></tr>
</table>
