# Supplementary material for: Identification of clustered microRNAs using an ab initio prediction method
Source: BMC Bioinformatics. 2005 Nov 7;6:267. doi: 10.1186/1471-2105-6-267 (PMC1315341; doi:10.1186/1471-2105-6-267)
Supplement: Additional File 9 — False negative, rat. Set of false negatives for rat, similar to the Additional file 7. Notice the unusual position of RN-4 in the secondary structure. [file 1471-2105-6-267-S9.pdf]

Name	Arm	Mature miRNA sequence	Cloning frequency	Pre-miRNA genomic coordinates	Pre-miRNA secondary structure	SVM score	Homologs (mismatches<6)	Other predictions
RN-1	5	ACUCAAAUGUGGGGCACACUU	1(m)	chr1, 64350964-64351026, -	G U - AC GAUU<br>GA ACUCAAA GUGG GGCAC UUCUG \<br>CU UGGGUUU CAUC UCGUG AAGAU A<br>G U A A- GCGU	-0.33	mmu-mir-295*(0)	L290
RN-2	3	ACCAGGAGGCUGAGGUCCCUU	1(m)	chr6, 134183482-134183559, +	U - U AUCCA A CU<br>GGUC CCU UGAGGGGCCUC GCCUCU GG UUAU U<br>CCGG GGA AUUCCCUGGAG CGGAGG CC AGUA U<br>C C U A---- - UU	-0.03	HN-4(1)<br>MN-6(0)	
RN-3	5	AUCAUGAUGGGCUCCUCGGUG	2(h), 1(m)	chr6, 134189465-134189537, +	GA----- UGA - C--- UC- AGG<br>UCA UGGGCU CCU GGUGU UCC \<br>GGU GCCCGA GGA CCACA AGG U<br>CUAGGAA UAG C AGUA CCA CGA	-1.81	hsa/mmu/rno-mir-433-3P(0)<br>MP-24(5)	
RN-4	5	GCUCGACUCAUGGUUUGAACC	4(m)	chr6, 134192432-134192513, +	U CUCU UG - CUC UC U<br>GUCGA UGA UGGUU AACCAC AG GAC AUGGU U<br>CAGCU ACU ACCAA UUGGUG UC CUG UACCA G<br>C ---- GU C AAA U- A	-0.06	mmu-mir-434(0)<br>MP-34(0)	
RN-5	5	AUAGUAGACCGUAUAGCGUAC	2(h), 4(m)	chr6, 134395538-134395603, +	AGA A AUA C UAU<br>GAG UAGU GACCGU GCGUACG UU \<br>CUC AUCA CUGGCA UGUAUGC AG C<br>CCA C CAA - UGU	-0.5	mmu-mir-411(0)<br>HN-5(0)	
	3	UAUGUAACACGGUCCACUAA	1(m)					
RN-6	5	AUGGUUGACCAUAGAACAUGC	1(m)	chr6, 134397179-134397245, +	A GUU GA C U<br>GA AAGAUG GACCAUA ACAUGCG UAC U<br>CU UUCUAC CUGGUAU UGUAUGC GUG C<br>C AC- GA U U	-0.81	hsa-mir-380(0)<br>mmu-miR-380(0)	
RN-7	3	GGCUGCAGCGUGAUCGCCUG	2(m)	chr6, 134403226-134403284, +	A-- - GAG - AG<br>AGCGGGC CG GCUGU AGCCCC CU G<br>UCGUCCG GU CGACG UCGGGG GA U<br>CUA G --- C CA	-2.11	MN-7(0)	
RN-8	3	AAUAUAACACAGAUGGCCUGU	3(h), 1(m)	chr6, 134431273-134431338, +	G UG A A C UAU<br>GAGGA AGGU UCUGUG UG GUUCG UU \<br>CUUUU UCCG AGACAC AU UAAGC AG U<br>G GU A A - UAA	-0.39	HP-49(0)<br>hsa-mir-410(0)<br>mmu-mir-410(0)	X26
RN-9	5	GAUGGAAACUGAGAGGA	1(m)	chr9, 22136907-22136975, +	U UU C A AAAC AG G<br>GGGG GGAGG UG UGG UGAGAGG GAUU \<br>CCCC CCUCC AC ACC ACUCUUC UUAG C<br>A U- - - C--- GU U	-2.01	hsa/mmu/rno-mir-184(4)	
RN-10	5	AGCAGCACACUGUGGUUUGUA	2(m)	chr10, 57087471-57087528, +	G C U -- AC<br>AGCA CACA UGUGGUUUG AC GGC U<br>UUGU GUGU ACACCAAAC UG CCG G<br>G C C CA GU	-0.06	hsa-mir-497(0)<br>HN-9(0)<br>MN-5(0)	X43
RN-11	5	CUUACAGUGCAGGUA	1(h)	chrX, 139814891-139814942, -	A G CU U<br>AAGUGCUU CAGUGCAG UAG UC U<br>UUCACGAA GUCACGUC AUC AG G<br>C - U- A	-0.22	hsa/mmu/rno-mir-17(0)<br>hsa/mmu/rno-mir-20(1)<br>hsa-mir-106a(0)<br>mm-mir-106a(1)	B971
